# Single‐cell sequencing combined with spatial transcriptomics reveals that the IRF7 gene in M1 macrophages inhibits the occurrence of pancreatic cancer by regulating lipid metabolism‐related mechanisms

**DOI:** 10.1002/ctm2.1799

**Published:** 2024-08-08

**Authors:** Ting Zhan, Yanli Zou, Zheng Han, XiaoRong Tian, Mengge Chen, Jiaxi Liu, Xiulin Yang, Qingxi Zhu, Meng Liu, Wei Chen, Mingtao Chen, Xiaodong Huang, Jie Tan, Weijie Liu, Xia Tian

**Affiliations:** ^1^ Department of Gastroenterology WuHan Third Hospital (Tongren Hospital of WuHan University) Wuhan China; ^2^ Department of Gastroenterology Zhongnan Hospital of Wuhan University Wuhan China

**Keywords:** exosomes, ILF3, IRF7, M1 macrophages, pancreatic adenocarcinoma, RPS18, scRNA‐seq, spatial transcriptomics

## Abstract

**Aim:**

The main focus of this study is to explore the molecular mechanism of IRF7 regulation on RPS18 transcription in M1‐type macrophages in pancreatic adenocarcinoma (PAAD) tissue, as well as the transfer of RPS18 by IRF7 via exosomes to PAAD cells and the regulation of ILF3 expression.

**Methods:**

By utilising single‐cell RNA sequencing (scRNA‐seq) data and spatial transcriptomics (ST) data from the Gene Expression Omnibus database, we identified distinct cell types with significant expression differences in PAAD tissue. Among these cell types, we identified those closely associated with lipid metabolism. The differentially expressed genes within these cell types were analysed, and target genes relevant to prognosis were identified. Flow cytometry was employed to assess the expression levels of target genes in M1 and M2 macrophages. Cell lines with target gene knockout were constructed using CRISPR/Cas9 editing technology, and cell lines with target gene knockdown and overexpression were established using lentiviral vectors. Additionally, a co‐culture model of exosomes derived from M1 macrophages with PAAD cells was developed. The impact of M1 macrophage‐derived exosomes on the lipid metabolism of PAAD cells in the model was evaluated through metabolomics analysis. The effects of M1 macrophage‐derived exosomes on the viability, proliferation, division, migration and apoptosis of PAAD cells were assessed using MTT assay, flow cytometry, EdU assay, wound healing assay, Transwell assay and TUNEL staining. Furthermore, a mouse PAAD orthotopic implantation model was established, and bioluminescence imaging was utilised to assess the influence of M1 macrophage‐derived exosomes on the intratumoural formation capacity of PAAD cells, as well as measuring tumour weight and volume. The expression of proliferation‐associated proteins in tumour tissues was examined using immunohistochemistry.

**Results:**

Through combined analysis of scRNA‐seq and ST technologies, we discovered a close association between M1 macrophages in PAAD samples and lipid metabolism signals, as well as a negative correlation between M1 macrophages and cancer cells. The construction of a prognostic risk score model identified RPS18 and IRF7 as two prognostically relevant genes in M1 macrophages, exhibiting negative and positive correlations, respectively. Mechanistically, it was found that IRF7 in M1 macrophages can inhibit the transcription of RPS18, reducing the transfer of RPS18 to PAAD cells via exosomes, consequently affecting the expression of ILF3 in PAAD cells. IRF7/RPS18 in M1 macrophages can also suppress lipid metabolism, cell viability, proliferation, migration, invasion and intratumoural formation capacity of PAAD cells, while promoting cell apoptosis.

**Conclusion:**

Overexpression of IRF7 in M1 macrophages may inhibit RPS18 transcription, reduce the transfer of RPS18 from M1 macrophage‐derived exosomes to PAAD cells, thereby suppressing ILF3 expression in PAAD cells, inhibiting the lipid metabolism pathway, and curtailing the viability, proliferation, migration, invasion of PAAD cells, as well as enhancing cell apoptosis, ultimately inhibiting tumour formation in PAAD cells in vivo. Targeting IRF7/RPS18 in M1 macrophages could represent a promising immunotherapeutic approach for PAAD in the future.

## Graphical abstract

This document is a detailed study on the role of IRF7 and RPS18 in regulating pancreatic cancer progression, specifically through the interactions between M1 macrophages and pancreatic cancer cells. The research utilises single‐cell RNA sequencing and spatial transcriptomics to identify the mechanisms by which IRF7 in M1 macrophages inhibits the occurrence of pancreatic cancer by regulating lipid metabolism‐related pathways. The study highlights the transfer of RPS18 via exosomes from M1 macrophages to pancreatic cancer cells, affecting the expression of ILF3 and thereby influencing the cancer cells' viability, migration, proliferation and apoptosis.

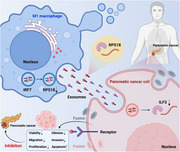

Graphical Headlights

This study utilised advanced single‐cell RNA sequencing technology.

The study revealed the regulatory role of IRF7 in M1 macrophages.

It identified the inhibitory effect of IRF7 on RPS18 transcription.

The study uncovered the mechanism by which IRF7 suppresses the growth of pancreatic adenocarcinoma cells.

## 1 INTRODUCTION

Pancreatic cancer (PC) is a prominent cause of cancer‐related mortality globally, exhibiting a low 5‐year survival rate. Pancreatic adenocarcinoma (PAAD) is the most prevalent type of PC, accounting for approximately 85%−90% of all PC.^1^, ^2^ It could be attributed to most patients being diagnosed at advanced stages, thereby having limited treatment alternatives.^3^ Recently, there has been an enhanced emphasis on comprehending the molecular mechanisms underlying PC to develop novel treatment modalities.^4^, ^5^, ^6^, ^7^ Macrophages, as a type of immune cell, are known to have a pivotal function in several types of cancer, including PC.^8^ Several subtypes of macrophages, including M1 and M2, play contrasting roles in cancer progression.^9^ The M1 type exhibits anti‐tumour functions, whereas the M2 type is associated with tumour‐promoting characteristics.^10^

Recent studies have elucidated the contribution of lipid metabolism to the initiation and progression of cancer, particularly in immune cells such as macrophages located within the tumour microenvironment.^11^ The advancement of single‐cell RNA sequencing (scRNA‐seq) and spatial transcriptomics (ST) technologies enables scientists to investigate the precise functions of macrophages in PC at the individual cell level.^12^ These technologies offer invaluable insights, enabling researchers to develop a comprehensive understanding of the intricate dynamics between macrophages and cancer cells and how these interactions influence cancer progression.^13^

Cellular communication within the tumour microenvironment, especially through exosomes (Exos), has been demonstrated to have a vital function in PC progression.^14^ Exos are diminutive vesicles capable of being transported from one cell to another, facilitating the transfer of a diverse array of bioactive molecules, including proteins, RNA and metabolites.^15^ This ability makes cells crucial in facilitating intercellular communication, particularly in cancer progression.^16^

Building upon the aforementioned background, this study aims to delve into how the lipid metabolism‐related gene IRF7 in M1 macrophages in PAAD regulates the transcription of RPS18 and influences the expression of ILF3 by delivering RPS18 to PAAD cells via Exos. We believe that a comprehensive understanding of this mechanism is not only crucial for the fundamental research of PAAD but also may offer valuable insights for the development of novel therapeutic approaches for this disease.

## 2 RESULTS

### 2.1 Single‐cell sequencing analysis reveals distinct cellular composition and alterations in PAAD and adjacent tissues

According to reports in relevant literature, the global annual incidence of PAAD has doubled over the past two decades. PC is uncommon in individuals under 40, and the likelihood of developing it rises with age. According to various surveys and projections, it is anticipated that the prevalence of PC will continue to rise in the upcoming decades. Therefore, studying the mechanisms of PC is of utmost importance to save patients' lives.^17^ The cellular alterations within the tumour microenvironment are pivotal in the malignant advancement of PC.^18^

**Key Points**

1. IRF7 and RPS18 regulation: IRF7 in M1 macrophages inhibits RPS18 transcription, reducing RPS18 transfer via exosomes to pancreatic cancer cells.
2. Impact on ILF3 expression: reduced RPS18 in pancreatic cancer cells leads to decreased ILF3 expression, affecting cancer cell functions.
3. Cancer cell inhibition: the downregulation of RPS18 and ILF3 results in decreased viability, migration and proliferation of pancreatic cancer cells, while promoting apoptosis.
4. Potential therapy: targeting the IRF7/RPS18 pathway in M1 macrophages offers a promising immunotherapeutic approach for pancreatic cancer.

To enhance our comprehension of the development process of PAAD and the alterations in the tumour microenvironment, we obtained cancer tissues (PAAD1‒3) and adjacent normal tissues (NOR1‒3) from three individuals with PAAD. Subsequently, we conducted single‐cell sequencing on these six tissue samples. Following the integration of sequencing data using the Seurat package, we conducted an initial analysis of various parameters, including the number of genes (nFeature_RNA), mRNA molecules (nCount_RNA) and the percentage of mitochondrial genes (percent.mt) in all cells of the scRNA‐seq data. The outcomes indicate that most cells exhibit nFeature_RNA values below 5000, nCount_RNA values below 20 000 and percent.mt values below 20% (Figure S1A). We conducted data quality control using 200 < nFeature_RNA < 5000 percent criteria.mt < 25. After removing low‐quality cells and duplicated genes, the research acquired a matrix containing expressions for 22 640 genes and 17 844 cells. The correlation calculation results indicate that the sequencing depth has a correlation coefficient (*r*) of −.03 with the filtered cell data, nCount_RNA and percent.mt. The correlation coefficient between nCount_RNA and nFeature_RNA is .69.

Furthermore, nCount_RNA has a correlation coefficient of −.02 percent.HB (Figure S1B). These findings discoveries that the quality of the screened cell data is satisfactory and could be utilised for further analysis.

**FIGURE 1:**
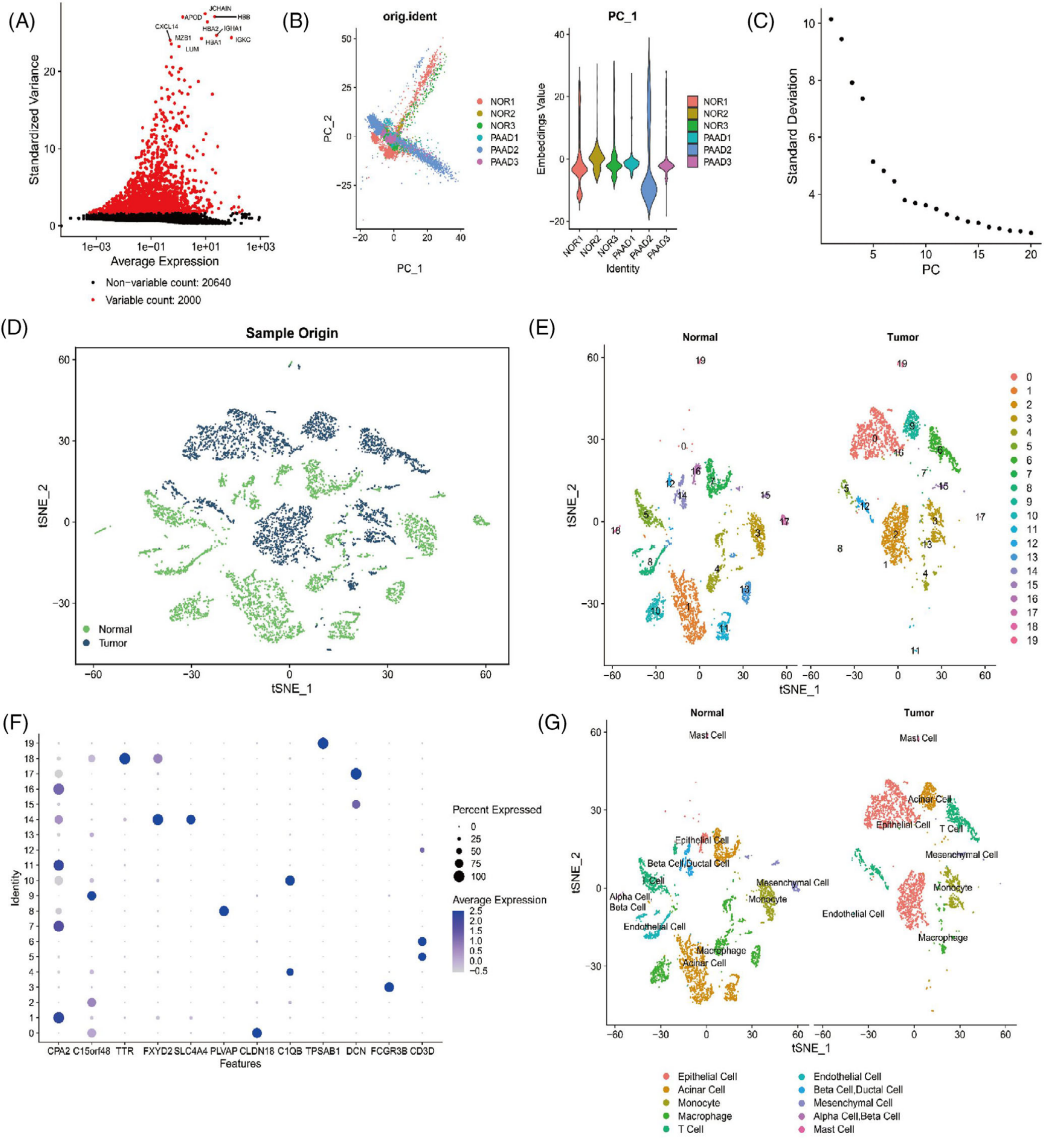
Cell clustering and annotation of single‐cell RNA sequencing (scRNA‐seq) data. (A) Differential gene expression analysis identified highly variable genes, with red representing the top 2000 highly variable genes and black representing genes with low variability. The top 10 genes in the highly variable gene set were labelled (*N* = 6). (B) Principal component analysis (PCA) analysis visualises the distribution of cells in PC_1 and PC_2, with each point representing a cell (*N* = 6). (C) Distribution of standard deviation of pancreatic cancers (PCs), with important PCs having a larger standard deviation (*N* = 6). (D) TSNE clustering visualisation shows the aggregation and distribution of cells from normal adjacent samples and pancreatic adenocarcinoma (PAAD) samples in two dimensions. Green represents normal adjacent samples, while deep blue represents PAAD samples. (E) TSNE clustering result visualises the aggregation and distribution of cells from normal adjacent tissue samples (normal, *N* = 3) and PAAD samples (tumour, *N* = 3), with each colour representing a cluster. (F) Expression patterns of known cell lineage‐specific marker genes in different clusters of normal adjacent tissue samples (normal, *N* = 3) and PAAD samples (tumour, *N* = 3). Deeper blue indicates higher average expression levels and larger circles indicate more cells expressing the gene. (G) Visualisation of cell annotation results based on TSNE clustering for the normal group (*N* = 3) and PAAD group (*N* = 3), with each colour representing a cell subtype.

Subsequent analysis was performed on the filtered cells, and highly variable genes were selected derived from the variance in gene expression. The leading 2000 genes with the highest variance were selected for subsequent analyses (Figure 1A). The cell cycle of the samples was determined applying the CellCycleScoring function (Figure S1C), and the data were preliminarily normalised. Subsequently, the data were reduced linearly based on the selected highly variable genes using principal component analysis (PCA). The PCA plot was then generated (Figure 1B). In this study, we provide the principal correlation heatmap illustrating the gene expression for PC_1 to PC_6 (Figure S1D). Furthermore, ElbowPlot was employed to arrange principal components (PCs) based on their standard deviation (Figure 1C). The results suggest that PC_1 to PC_14 effectively capture the details related to the selected highly variable genes and hold substantial analytical importance.

Next, we implemented the t‐SNE algorithm to decrease the dimensionality of the first 14 PCs non‐linearly. Subsequently, cluster analysis was conducted using a resolution of .2 (Figure S2). We performed cluster analysis and obtained 20 clusters. Subsequently, we determined the expression profile of marker genes in each cluster (Figure 1D,E). Through a thorough literature search, we identified known marker genes that are specific to different cell lineages. The cells were then annotated using the online resource CellMarker (Figures 1F and S1E). A total of 11 cell types have been identified, including acinar cell, alpha cell, beta cell, ductal cell, endothelial cell, epithelial cell (cancer cell), macrophage, mast cell, mesenchymal cell, monocyte and T cell (Figure 1G). Among the identified clusters, clusters 1, 7, 9 and 11 represent acinar cells; cluster 18 corresponds to alpha cells and beta cells; and cluster 14 encompasses beta cells and ductal cells. Additionally, cluster 8 is composed of endothelial cells; clusters 0, 2 and 16 consist of epithelial cells (cancer cells); clusters 4, 10 and 13 consist of macrophages; cluster 19 represents mast cells; clusters 15 and 17 consist of mesenchymal cells; cluster 3 corresponds to monocytes; clusters 5, 6 and 12 are characterised as T cells. In PAAD tissue, the acinar cell, macrophage and endothelial cells demonstrate reduction compared to normal samples adjacent to cancer. Conversely, the epithelial cell (cancer cell) exhibits an increase. Among the cell types found in tumour tissue, acinar cells, epithelial cells (cancer cells) and macrophages exhibit the highest proportions of cellular content.

The results above suggest that the PAAD samples could be classified into 20 clusters, including their adjacent normal samples. These clusters collectively consist of 11 different cell subtypes. The PAAD organisation exhibits a noticeable decline in acinar cells, macrophages and endothelial cells, with a surge in epithelial cells (cancer cells).

### 2.2 Macrophage‒lipid metabolism interplay and its influence on pancreatic ductal adenocarcinoma progression

**FIGURE 2:**
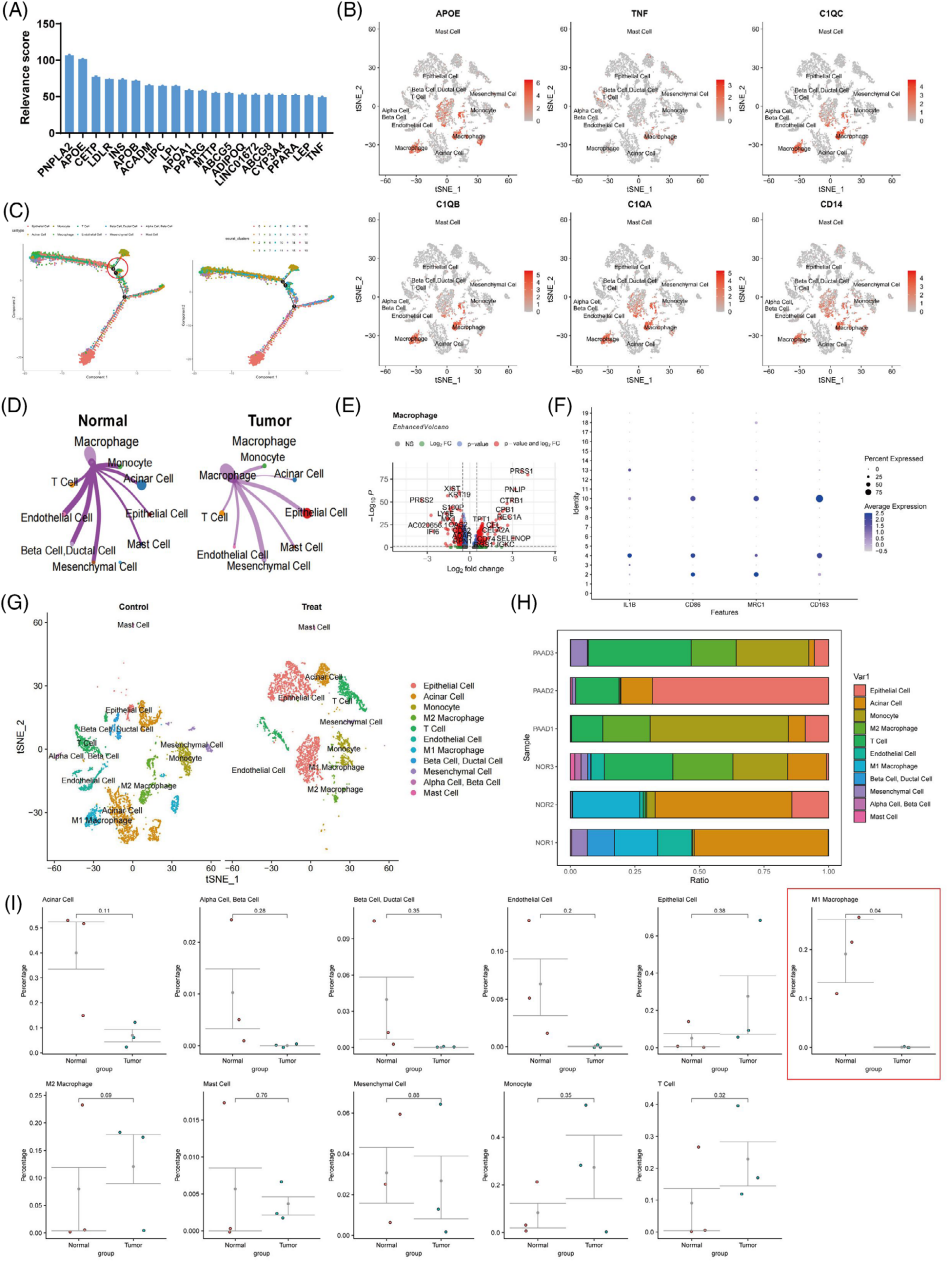
Study on the differential molecular characterisation of macrophages in pancreatic cancer tissues. (A) The relevance scores of the top 20 genes in the ‘LIPID METABOLIC PROCESS’ gene set in the Genecards database. (B) Expression profiles of macrophage marker genes in different cell subpopulations in adjacent normal tissues and pancreatic adenocarcinoma (PAAD) tissues samples (*N* = 6), with darker red indicating higher average expression levels. (C) Construction of cell trajectories based on cell types and clustering (*N* = 6). (D) Differential cell‒cell communication map of macrophages in adjacent normal tissue samples compared to PAAD tissue samples, where line thickness represents interaction strength (*N* = 6). (E) Volcano plot of differentially expressed genes between macrophages in adjacent normal tissue samples and PAAD samples, with red dots on the left of the dashed line indicating genes with higher expression in PAAD samples and dots on the right indicating genes with lower expression in PAAD samples (*N* = 6). (F) Expression profiles of known M1 macrophage‐specific marker genes in different clusters in adjacent normal tissue (normal, *N* = 3) and PAAD samples (tumour, *N* = 3), with darker blue indicating higher average expression levels and larger circles representing more cells expressing that gene. (G) Visualisation of cell annotations based on t‐SNE clustering for normal group (*N* = 3) and PAAD group (*N* = 3), with each colour representing a cell subpopulation. (H) Proportion of different cell subpopulations in each sample, represented by different colours (*N* = 6). (I) Differences in cell composition between adjacent normal tissue and PAAD samples analysed by *t*‐test, with control (*N* = 3) representing adjacent normal tissue samples, treat (*N* = 3) representing PAAD tissue samples, and *p*‐values indicating the significance level of differences in cell composition between control and treat groups.

Numerous studies have demonstrated the critical involvement of lipid metabolism in the pathogenesis and advancement of pancreatic ductal adenocarcinoma (PDAC). The metabolism of lipid synthesis has been demonstrated to advance the propagation and growth of PAAD cells.^19^, ^20^, ^21^ To examine the correlation between lipid metabolism pathways and PAAD, we retrieved a gene set associated with the ‘LIPID METABOLIC PROCESS’ from the Genecards database. Then, we calculated the relevance scores for the top 20 genes (Figure 2A). Existing literature demonstrates that the influence of immune microenvironments, such as macrophages, drives the reprogramming of lipid metabolism in cancer cells.^22^ The results indicate that APOE, the second‐ranked gene, is a macrophage marker gene. Therefore, we propose that macrophages are closely associated with lipid metabolism signalling and influence the occurrence and progression of PAAD (Figure 2A).

To validate this hypothesis, we initially confirmed the annotation of macrophages (Figure 2B), which yielded accurate results. To identify key cellular players, we performed pseudotime analysis using the ‘monocle’ package, sorting cells and constructing trajectories based on gene expression trends (Figure 2C). We observed a substantial presence of macrophages at branch points 1 and 3, suggesting their potential significant role in the tumour microenvironment of PAAD by possibly promoting the differentiation of other immune cells. Subsequently, to investigate functional variances of macrophages between adjacent normal tissues and PAAD samples, the study employed the R package ‘CellChat’ for pathway activity exploration among various cell types. The discoveries demonstrated a tighter interplay between macrophages and epithelial cells (cancer cells) in the PAAD tissue in a comparison of adjacent normal tissues, with enhanced interactions (Figures 2D and S3A,B).

Furthermore, the investigation carried out distinct examination of gene expression in macrophages between adjacent normal tissue and PAAD samples, recognising 130 notably upregulated genes and 144 markedly downregulated genes with |log_2_ FC| > .585 and *p*‐value < .05 (Figure 2E). Gene enrichment analysis unveiled that the upregulated genes in PAAD samples were predominantly enriched in biological processes such as protein targeting to ER, protein targeting to the membrane and viral transcription, while the downregulated genes were chiefly associated with immune system processes, immune responses and innate immune responses (Figure S3C,D). According to the findings from Kyoto Encyclopedia of Genes and Genomes (KEGG) enrichment analysis, the upregulated genes in PAAD samples demonstrated significant enrichment in signalling pathways associated with pancreatic secretion, protein absorption and lipid assimilation, while the downregulated genes showed enrichment in pathways such as apoptosis, the PPAR signalling pathway and lipid metabolism (Figure S3E,F).

Subsequently, we annotated macrophage subtypes based on marker genes, distinguishing them into M1‐type and M2‐type macrophages (Figure 2F,G). Using Seurat analysis, the research computed the relative abundance of various cell categories in each individual specimen (Figure 2H) and conducted *t*‐tests to analyse differences in cell types between adjacent normal and PAAD tissue samples. Our analysis revealed a decrease in M1‐type macrophage content in PAAD tissues relative to adjacent normal tissues (Figure 2I).

In conclusion, differing from adjacent normal tissues, PAAD samples exhibit a stronger interaction with epithelial cells (cancer cells), with enhanced connectivity and a reduction in M1‐type macrophage content. Given their close association with lipid metabolism signalling, macrophages likely hold a pivotal position in the PAAD tumour microenvironment by potentially promoting the differentiation of other immune cells.

### 2.3 ST reveals intense cell‒cell interactions and macrophage influence in PAAD tumour microenvironment

In recent years, the integration of ST and scRNA‐seq technologies has been employed to characterise the spatial composition of diverse cell types within tissues. This integration addresses the spatial information loss often associated with scRNA‐seq and holds great potential for applications in the field of biology.^23^ The SPOTlight package leverages the combination of ST data and scRNA‐seq data to infer the spatial distribution of different cell types and states within tissues, facilitating the analysis of their interactions.^24^

To further investigate the arrangement of various cell types in PAAD tissue, we employed the ST technique to analyse frozen sections of cancer tissue obtained from three additional PAAD patients. It allowed for an unbiased mapping of the expressed transcripts within the tissue. We initially analysed the number of genes (nFeature_Spatial), the count of mRNA molecules (nCount_Spatial) and the percentage of mitochondrial genes (percent.mt) in all cells of the ST data using the Seurat package for data integration. The findings suggested that the majority of cells displayed nFeature_Spatial values below 10 000, nCount_Spatial below 50 000 and percent.mt below 20% (Figure S4A). After filtering, the correlation calculations for sequencing depth exposed that the correlation coefficient (*r*) between nCount_Spatial and percent.mt was NA.

On the other hand, the correlation coefficient between nCount_Spatial and nFeature_Spatial was .91, and the correlation coefficient between nCount_Spatial and percent.HB was −.13 (Figure S4B). This result indicates that the short‐term (ST) study's data exhibits high quality and is appropriate for additional analysis. Figure S4C displays the distribution of nCount_Spatial across various organisational slices. The sample cell cycle was assessed using the CellCycleScoring function (Figure S4D), and the data were standardised (Figure S4E).

**FIGURE 3:**
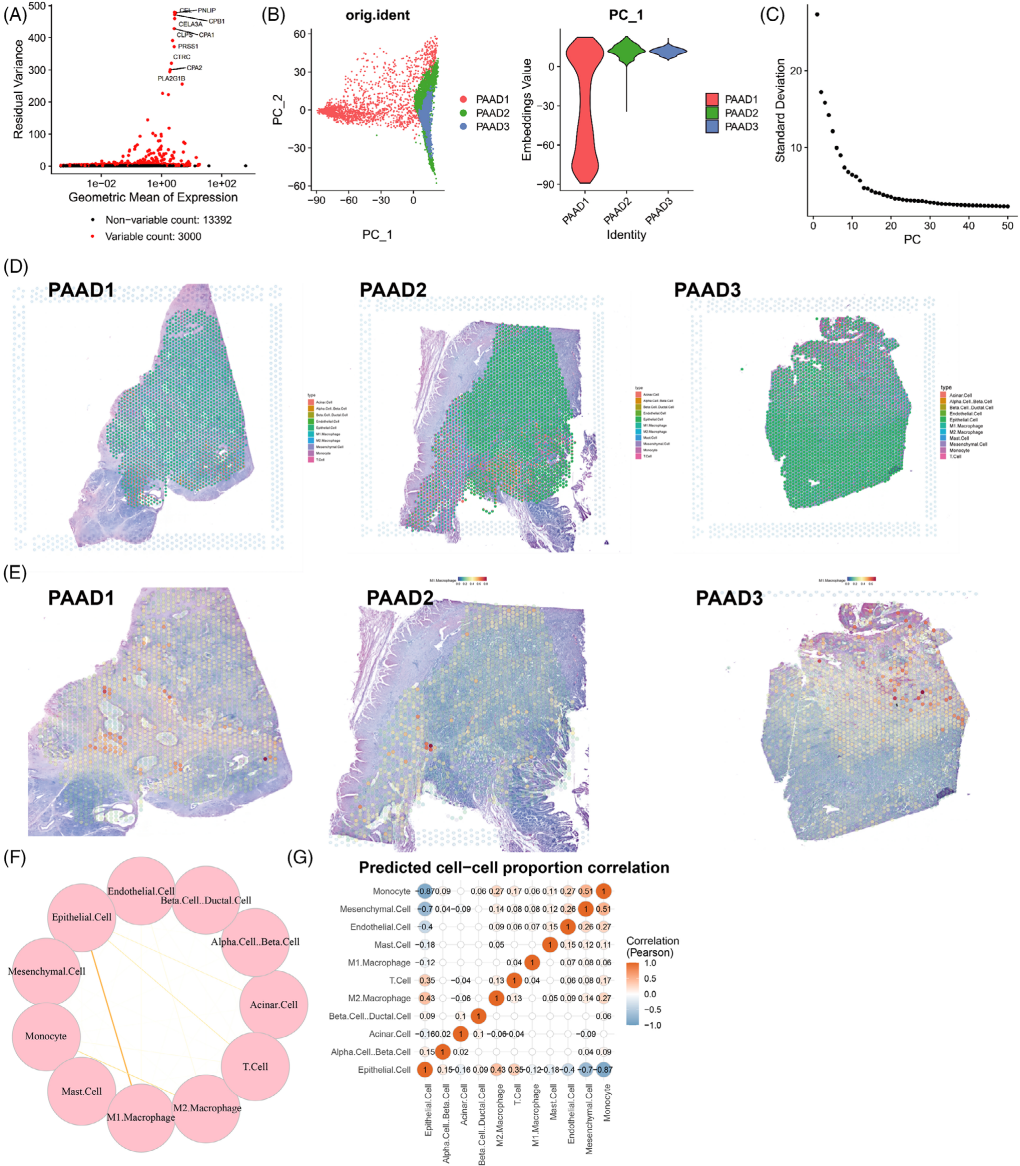
Analysis results combining single‐cell RNA sequencing (scRNA‐seq) and spatial transcriptomics (ST). (A) Differential gene expression analysis identified highly variable genes, with red representing the top 3000 highly variable genes and black representing genes with low variability. The top 10 genes in the highly variable gene set were labelled (*N* = 3). (B) Principal component analysis (PCA) analysis visualises the distribution of cells in PC_1 and PC_2, with each point representing a cell (*N* = 3). (C) Distribution of standard deviations of pancreatic cancers (PCs), with important PCs having a larger standard deviation (*N* = 3). (D) Distribution of different cell types on ST slices from pancreatic adenocarcinoma (PAAD) tissues (*N* = 3), represented by pie charts showing the proportion of different cell types in each spot. (E) Distribution of M1 macrophages in PAAD tissue sections on Expressed Sequence Tag (EST) data (*N* = 3), with the colour getting redder indicating a higher proportion of M1 macrophages in that spot. (F) Circular graph showing interaction intensity between different cells based on ST data, with thicker lines indicating stronger interactions (*N* = 3). (G) Heatmap of cell correlations based on ST data, with numerical values indicating correlation coefficients between cells (*N* = 3).

Further analysis was performed on the ST data. Genes with high expression variance were filtered, and the top 3000 genes based on variance were selected for subsequent analysis (Figure 3A). Subsequently, we conducted PCA to linearly decrease the data's dimensionality and produced a PCA plot (Figure 3B). Furthermore, an ElbowPlot was employed to rank the PCs based on their standard deviation (Figure 3C). In addition, we presented the heatmap depicting the major correlated gene expression for PC_1 to PC_6 (Figure S4F). The results suggest that PC_1 to PC_14 could effectively document the data from the chosen highly variable genes and possess considerable analytical importance.

Next, we employ the T‐Distributed Stochastic Neighbor Embedding (TSNE) algorithm to nonlinearly lower the dimensionality of the first 30 PCs and conduct cluster analysis using a resolution of .4. To determine the enrichment of specific cell types in a particular tissue region, we assessed the degree of overlap between genes mapped to that region and genes specific to each cell type identified by scRNA‐seq data. Based on this analysis, we performed cell annotation on the ST data (Figure 3D). The distribution of M1‐type macrophages is depicted in Figure 3E. Leveraging scRNA‐seq data, the ‘SPOTlight’ package in R was utilised to obtain information on cell interactions in spatial context. Circular plots illustrating the strength of cell‒cell interactions (Figure 3F) and a heatmap of cell correlations (Figure 3G) were generated. The results indicate that in the PAAD tumour microenvironment, the interaction strength between epithelial cells (cancer cells) and M1‐type macrophages is most significant. Moreover, these interactions exhibit a negative correlation with both cell types, consistent with previous literature findings. The differing functions of monocyte‐derived macrophages in maintaining normal tissue balance and promoting tumour growth could be potentially elucidated by their distinct characteristics. Macrophages demonstrate functional plasticity, allowing them to alter their polarisation states to adapt to varying physiological conditions. Specifically, M1‐type macrophages generate type I pro‐inflammatory cytokines, engage in antigen presentation and possess anti‐tumour effects. On the contrary, M2‐type macrophages create type II cytokines, promote anti‐inflammatory responses and exhibit pro‐tumour functionality. Additionally, stromal cells in the tumour microenvironment typically foster tumour growth and metastasis and are often targeted for cancer therapy.^25^

In summary, in PAAD samples, there exists a close association between M1‐type macrophages and epithelial cells (cancer cells), showing a negative correlation. These interactions contribute to the suppression of cancer cells within the PAAD tumour microenvironment.

### 2.4 IRF7 modulates prognosis in PAAD by regulating RPS18 expression in M1 macrophages

**FIGURE 4:**
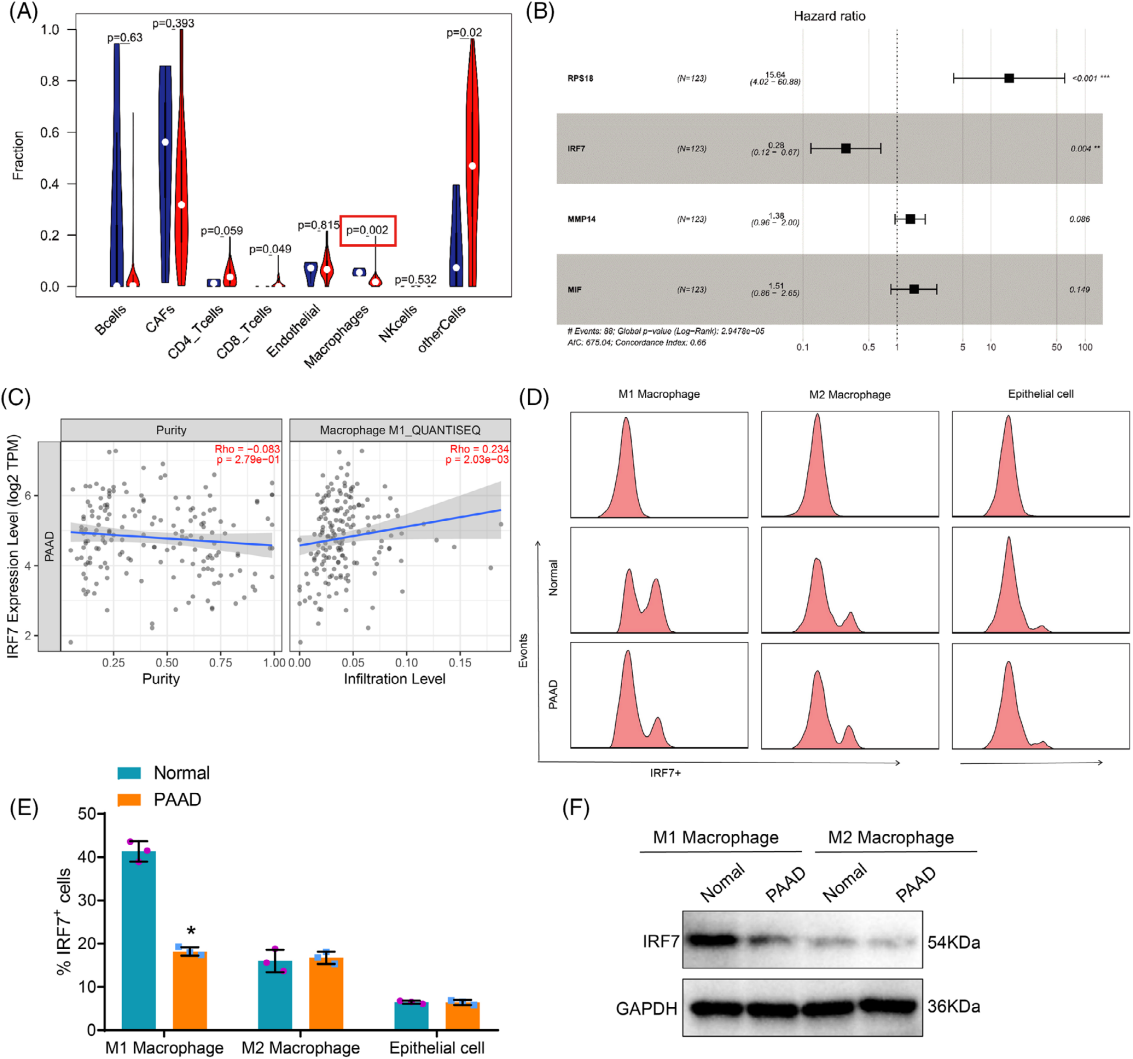
Construction of a lipid metabolism marker gene‐based prognostic risk scoring model using the Gene Expression Omnibus (GEO) database. (A) Relationship between pancreatic adenocarcinoma (PAAD) and tumour‐infiltrating immune cells based on TCGA PAAD transcriptome data (*N* = 183), with blue representing the control group and red representing the experimental group. (B) The forest plot of the Cox risk scoring model shows the model genes and their risk coefficients. (C) Immune infiltration analysis using TIMER v2.0 database. (D) Separation of M1 and M2 macrophages in different tissues by flow cytometry. (E) Quantitative statistics of flow cytometry. (F) Western blot analysis of IRF7 expression in M1 and M2 macrophages isolated from different tissues. Asterisk (*) indicates *p* < .05 compared to the normal group, with all cell experiments repeated three times.

To confirm the findings above, we conducted an immune infiltration analysis using the transcriptome data of PAAD obtained from the TCGA database. The results demonstrated a decrease in the infiltration score of macrophages in PAAD tissue samples compared to normal tissue samples (Figure 4A). The results of the immune infiltration analysis also revealed a notable decrease in the levels of macrophage immune infiltration in PAAD tissue samples.

To examine the influence of macrophages on the occurrence and prognosis of PAAD, we isolated differentially expressed genes (DEGs) in macrophages and developed a predictive risk‐scoring model. The transcriptome dataset GSE183795, relevant to PAAD, was downloaded from the Gene Expression Omnibus (GEO) database. From this dataset, we extracted the expression levels of 274 DEGs. The samples are randomly divided into ‘Train’ and ‘Test’ parts to construct and validate the model. After extracting genes and grouping samples, we conducted a univariate Cox analysis on the expression matrix of the Train group to identify genes associated with patient prognosis. We identified a total of nine genes that are associated with the prognosis of patients with PAAD (Figure S5A).

Subsequently, we utilise the ‘glmnet’ package to implement Lasso regression and mitigate model overfitting. Following Lasso regression, the results indicate that choosing eight genes for model construction yields the most optimal simulation effect, as illustrated in Figure S5B,C. Lasso regression identified several genes, including KRT17, RPS18, INS, IRF7, RPS29, SRGAP1, MMP14 and MIF. Using the gene expression data from the Train group, we employed the ‘Survival’ package to construct a Cox model with expressed genes chosen through Lasso regression. The resultant model yields hazard coefficients for the genes and risk scores for each sample. After constructing the model, we developed a prognostic risk‐scoring model consisting of four genes: RPS18, IRF7, MMP14 and MIF. Figure 4B depicts the risk coefficients associated with each gene. The *p*‐values of MMP14 and MIF are greater than .05. Genes RPS18 and IRF7 demonstrated significance at *p* < .05. However, only IRF7 is classified as a low‐risk gene. The survival analysis results for four genes are presented in Figure S5A‒D. RPS18 demonstrates an unfavourable effect on patient survival with high expression, while IRF7, MMP14 and MIF exhibit a favourable impact with high expression, contradicting the prognosis risk score results. The expression levels of four genes in macrophages from single‐cell sequencing samples are displayed in Figure S5D, with IRF7 showing the most difference. Analysis of immune infiltration using TIMER v2.0 database revealed a positive correlation between IRF7 and the infiltration levels of M1 macrophages, while showing a negative correlation with tumour cell purity (Figure 4C).

To examine the expression profile of IRF7 in macrophages infiltrating tumours, we performed flow cytometry analysis to quantify the amounts of IRF7+ M1 and IRF7+ M2 macrophages in adjacent normal tissues and PAAD tissues. The results demonstrated that the level of IRF7+ M1 polarised macrophages in normal tissues adjacent to cancer was markedly higher than in PAAD tissues, whereas the content of IRF7+ M2 polarised macrophages exhibited no alteration (Figure 4D,E). Subsequently, immune cells were purified from PAAD tissues using CD86 and CD163 beads. The content of IRF7 was then determined in both cell types using Western blot analysis. The outcomes illustrated that the level of IRF7 changed exclusively in M1 macrophages (Figure 4F). Thus, we chose M1 macrophages as the focus of our subsequent analysis.

After analysing the results above, we detected genes differentially expressed in macrophages and developed a risk‐scoring model, which identified the low‐risk gene IRF7.

### 2.5 IRF7 modulation in M1 macrophages alters lipid metabolism and suppresses PAAD cell proliferation, viability and migration

Multiple studies have demonstrated that modulating lipid metabolism could influence the biological functions of cancer cells and stimulate cancer progression.^26^, ^27^, ^28^, ^29^ Macrophages could influence cellular lipid metabolism processes.^30^ To investigate the impact of IRF7+ M1 macrophages on the lipid metabolism pathways and biological functions of PAAD cells, we utilised CRISPR/Cas9 gene editing technology to create IRF7‐knockout (IRF7‐KO) M1 macrophages (IRF7‐WT as the wild‐type control for IRF7‐KO). RT‐qPCR and Western blot analyses were applied to assess the expression levels of IRF7 and RPS18 in single‐cell clones, with selection of single‐cell clones demonstrating zero IRF7 expression for subsequent expansion (Figure S6A). Interestingly, IRF7‐KO cells exhibited significantly increased RPS18 expression levels (Figure S6B,C) and enhanced stability of RPS18 mRNA (Figure S6D). Additionally, we established M1 macrophages overexpressing IRF7 through lentivirus transduction (oe‐IRF7, with oe‐NC as the control) and validated the transfection efficiency of IRF7 and expression levels of RPS18 using RT‐qPCR and Western blot (Figure S6E). Notably, it was demonstrated by the results that the changes in RPS18 expression levels in oe‐IRF7 cells were opposite to those in IRF7‐KO cells (Figure S6F,G), and there was a significant reduction in the stability of RPS18 mRNA (Figure S6H).

**FIGURE 5:**
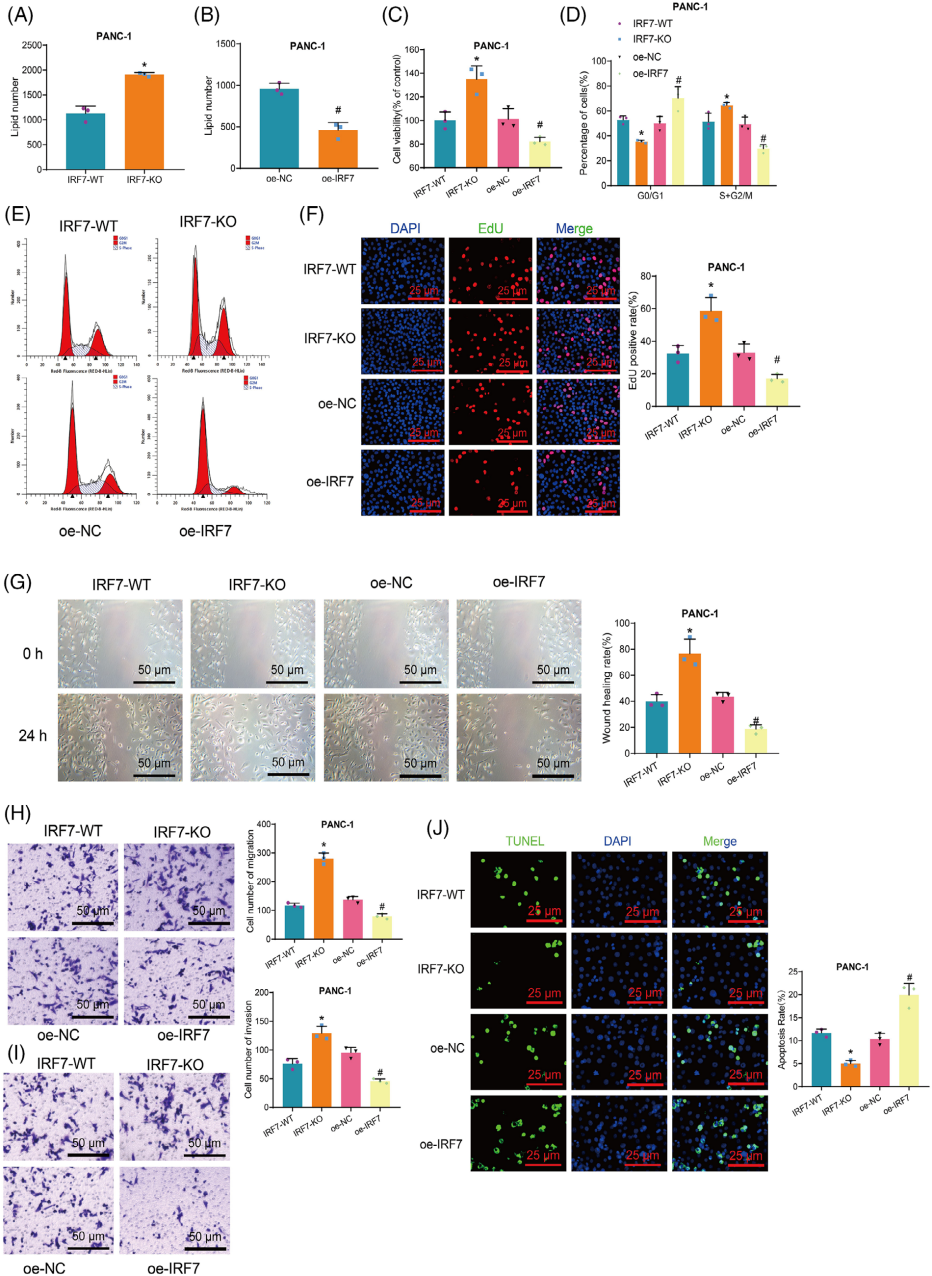
Impact of IRF7 on the biological functions of pancreatic adenocarcinoma (PAAD) cells. (A and B) Metabolomic analysis of each group's total lipid content in PANC‐1 cells. (C) MTT assay to measure cell viability in each group of PANC‐1 cells. (D) Statistical analysis of flow cytometry results. (E) Flow cytometry analysis of cell cycle changes in PANC‐1 cells in each group. (F) EdU assay to detect the proliferative capability of PANC‐1 cells in each group (scale bar: 25 µm). (G) Scratch assay to evaluate the migration ability of PANC‐1 cells in each group (scale bar: 50 µm). (H and I) Transwell assay to measure the migration and invasion ability of PANC‐1 cells in each group (scale bar: 50 µm). (J) TUNEL assay to determine the apoptosis rate of PANC‐1 cells in each group (scale bar = 25 µm). Asterisk (*) indicates *p* < .05 compared to the IRF7‐WT group and symbol (#) indicates *p* < .05 compared to the oe‐NC group. Cell experiments were repeated three times.

Next, we co‐cultured extracellular vesicles from different groups of M1 macrophages with PAAD cell lines (PANC‐1/BxPC‐3). We then quantified the total content of lipid metabolites in PAAD cells post co‐culturing using metabolomics analysis. The results revealed a substantial growth in the total content of lipid metabolites in PANC‐1 and BxPC‐3 cells in the IRF7‐KO group compared to the IRF7‐WT group (Figures 5A and S7A). Conversely, a notable reduction in the total content of lipid metabolites was observed in PANC‐1 and BxPC‐3 cells in the oe‐IRF7 group contrasting with the oe‐NC group (Figures 5B and S7B).

Moreover, we assess cell viability applying the 3‐(4,5‐dimethylthiazol‐2‐yl)‐2,5‐diphenyltetrazolium bromide (MTT) assay, cell division and proliferation capacity through flow cytometry and 5‐Ethynyl‐2′‐deoxyuridine (EdU) experiments, cell migration and invasion capacity using wound scratch and Transwell assays, and cell apoptosis using Terminal Deoxynucleotidyl Transferase dUTP Nick End Labeling (TUNEL) staining procedures. The results demonstrated that compared to the IRF7‐WT group, the IRF7‐KO group exhibited increased cell viability, proliferation and migration capabilities. Moreover, there was an elevation in the proportion of cells in the S+G2/M phase accompanied by a decrease in cell apoptosis. Compared to the oe‐NC group, the oe‐IRF7 group demonstrated diminished cellular viability, proliferation and migratory capacities, along with a decline in the percentage of cells in the S+G2/M phase, and an increase in cell apoptosis levels (Figures 5C‒J and S7C‒J).

The results above indicate that IRF7 could hinder lipid metabolism in M1 macrophages, suppressing cell viability, proliferation, migration, invasion and promoting apoptosis in PAAD cells.

### 2.6 IRF7‐deficient M1 macrophage‐derived Exos transport RPS18 to PAAD cells, modulating ILF3 expression

**FIGURE 6:**
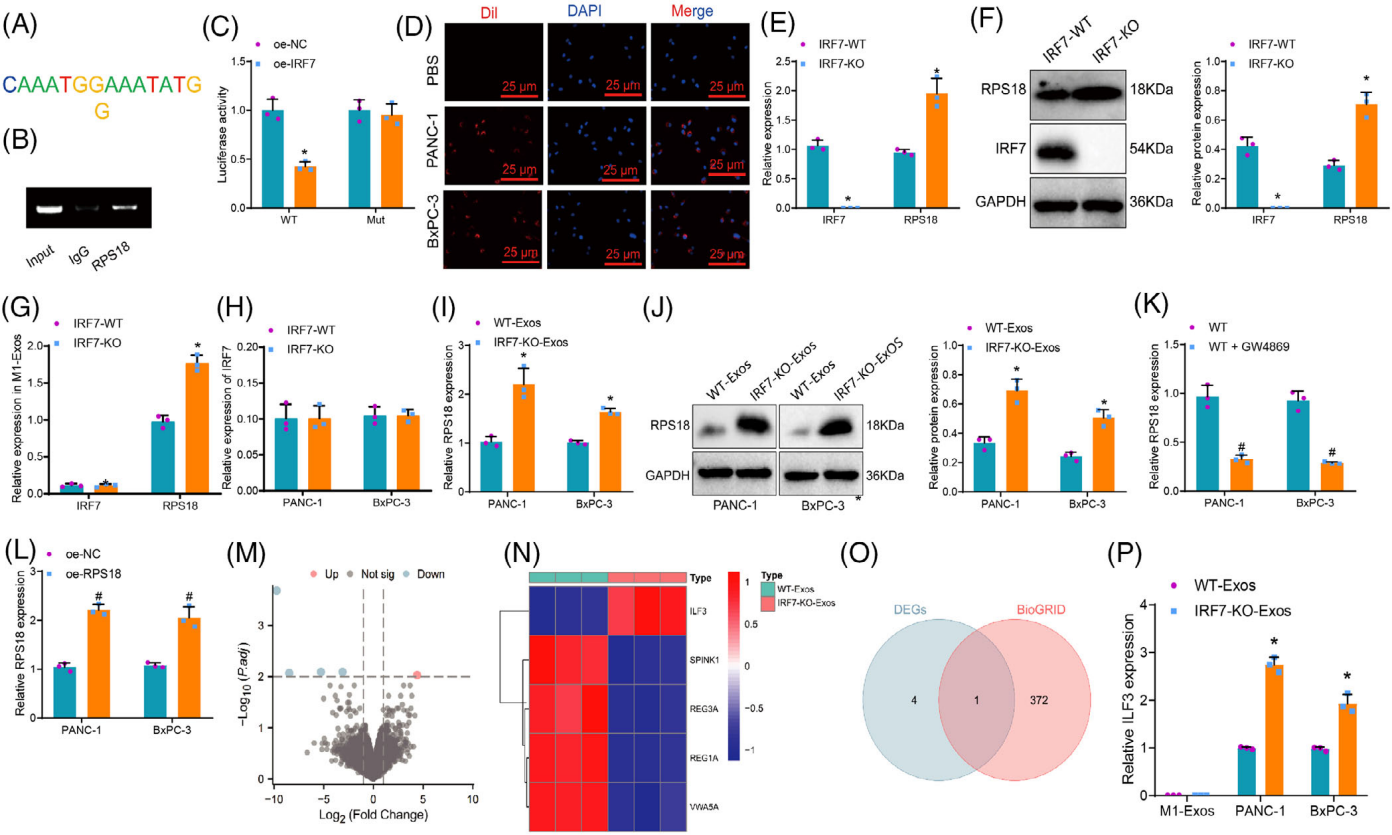
Selection of downstream target genes regulated by IRF7/RPS18 transferred by M1‐Exos to pancreatic adenocarcinoma (PAAD) cells. (A) Using LASAGNA‐Search 2.0 to predict the binding sites of the transcription factor IRF7 with the target gene RPS18. (B) Observing the content of amplification products in ChIP‐qPCR in M1 macrophages by agarose gel electrophoresis. (C) Analysing the activity of the RPS18 promoter‐driven luciferase in HEK293T cells using the luciferase reporter gene method (*N* = 3). Asterisk (*) indicates *p* < .05 compared to the pGL3‐basic + IRF7 group, and the cell experiments were repeated three times. (D) Observing the uptake of M1‐Exos by PANC‐1/BxPC‐3 cells using laser scanning confocal microscopy, with red fluorescence representing Dil and blue fluorescence representing DAPI nuclear staining (scale bar: 50 µm). (E and F) Detecting the gene expression of IRF7 and RPS18 in various groups of M1 macrophages using Western blot and RT‐qPCR. (G) Detecting the gene expression of IRF7 and RPS18 in various groups of M1‐Exos using RT‐qPCR. (H) Detecting the gene expression of IRF7 in various groups of PAAD cells using RT‐qPCR. (I and J) Detecting the gene expression of RPS18 in various groups of PANC‐1/BxPC‐3 cells using RT‐qPCR and Western blot. (K and L) RT‐qPCR was used to detect the expression level of RPS18 in PAAD cells co‐cultured in different groups. (M) Volcano plot showing differentially expressed mRNA between PANC‐1 cells co‐incubated with three WT‐Exos and three IRF7‐KO‐Exos in high‐throughput sequencing data. (N) Heatmap revealing the differential expression of five genes in sequencing data, with WT‐Exos (*N* = 3) and IRF7‐KO‐Exos (N = 3) representing samples of M1 macrophages secreted Exos co‐cultured before and after IRF7‐knockout with PAAD cells. (O) Venn diagram showing the intersection between differentially expressed genes in sequencing data and genes predicted by BioGRID to interact with RPS18. (P) Detecting the gene expression of ILF3 in various groups of M1‐Exos and PANC‐1/BxPC‐3 cells using RT‐qPCR. Asterisk (*) indicates *p* < .05 compared to the IRF7‐WT or WT‐Exos group, asterisk (*) indicates *p* < .05 compared to the IRF7‐WT group and symbol (#) indicates *p* < .05 compared to the WT group or oe‐NC group, with cell experiments repeated three times.

IRF7, a transcription factor belonging to the interferon regulatory factor (IRF) family, has previously been identified as a tumour suppressor across various cancer forms. It functions by suppressing the transcription levels of its target genes, thereby playing an inhibitory role in cancer development.^31^ The LASAGNA‐Search 2.0 database prediction results demonstrate that IRF7 can bind to the RPS18 promoter (Table S1), thereby regulating its transcriptional activity. The binding sites are illustrated in Figure 6A, and the correlation analysis between IRF7 and RPS18 in TCGA‐PAAD data are shown in Figure S8A. Initially, we employed gel electrophoresis to present the binding sites detected by CHIP‐qPCR. The results demonstrated the presence of binding sites within the promoter region of RPS18 in the M1 macrophage model, as shown in Figure 6B. Subsequently, we co‐transfected IRF7‐WT plasmid along with a fluorescent reporter plasmid containing the binding site of the RPS18 promoter, or co‐transfected IRF7‐WT plasmid with a fluorescent reporter plasmid containing a mutated binding site of the RPS18 promoter. Luciferase assay results consistently indicated that overexpression of the transcription factor IRF7 in the M1 macrophage model inhibited the expression of the target gene RPS18. However, when the binding site within the promoter region of RPS18 was mutated, the inhibitory effect of IRF7 was significantly diminished. This finding suggests that IRF7 does inhibit the expression of RPS18 by binding to its promoter, as illustrated in Figure 6C.

Research indicates that Exos are crucial in facilitating communication between cancer and non‐cancer cells. Exosomes originating from M1‐type macrophages could impact the expression of cancer‐related genes by transmitting RNA molecules, thereby playing a role in cancer inhibition.^32^, ^33^ Hence, it is hypothesised that IRF7 hampers the transcription process of RPS18 and facilitates the transportation of RPS18 to PAAD cells via Exos derived from M1 macrophages, subsequently influencing its biological functions.

To confirm the above speculation, we cultured M1‐type macrophages and collected the exosomes (M1‐Exos) secreted by these macrophages. The results revealed that the Exos, which were isolated, demonstrated a particle size distribution primarily focused within the 100–150 nm range. Moreover, the Exos exhibited elevated levels of CD9, CD63 and CD81 proteins, known as exosome marker proteins. Conversely, the levels of calnexin, a cell marker protein, were found to be low. Additionally, other impurities were detected (Figure S8B‒D). After labelling the separated Exos with Dil, they were co‐cultured with PANC‐1/BxPC‐3 cells for 24 h. The uptake of Exos by PANC‐1/BxPC‐3 cells was observed using a laser scanning microscope. The results demonstrated the absence of a green fluorescence signal in the PANC‐1/BxPC‐3 cells subjected to phosphate‐buffered saline (PBS) treatment, whereas a distinct red fluorescence signal was detected in the PANC‐1/BxPC‐3 cells exposed to Exos (Figure 6D). The results above suggest that PAAD cells could internalise Exos released from M1 macrophage. Initially, in M1 macrophages, knocking out IRF7 resulted in a significant increase in RPS18 expression as revealed by protein immunoblotting and qPCR analysis in the IRF7‐KO group (Figure 6E,F). Subsequently, after extracting the corresponding M1‐Exos, qPCR analyses indicated a significant increase in RPS18 expression in M1‐Exos of the IRF7‐KO group compared to the IRF7‐WT group. However, there was no significant change in the expression of IRF7 in M1‐Exos and PAAD cells (Figure 6G,H). These findings suggest that RPS18 enriched within M1 macrophages of the IRF7‐KO group is transferred to the Exos. Finally, when co‐culturing the obtained M1‐Exos with PANC‐1/BxPC‐3 cells, Western blot and RT‐qPCR results demonstrated a considerable boost in RPS18 expression in PANC‐1/BxPC‐3 cells of the IRF7‐KO‐Exos group when juxtaposed with the WT‐Exos group after cellular uptake of M1‐Exos (Figure 6I,J). When M1 macrophages were co‐cultured with PAAD cells and treated with GW4869, the RPS18 content in PAAD cells notably decreased upon treatment of M1 macrophages with GW4869, providing evidence that RPS18 can transferred from M1 macrophages to PAAD cells via extracellular vesicles secretion (Figure 6K). Additionally, overexpression of RPS18 in M1 macrophages through lentiviral transduction (Figure S8E) led to a significant upregulation of RPS18 content in PAAD cells co‐cultured with the oe‐RPS18 group contrasting with the oe‐NC group, pointing out that alterations in RPS18 expression in M1 macrophages result in changes in RPS18 expression in PAAD cells (Figure 6L).

Subsequently, to elucidate the downstream target genes affected by M1 macrophage‐derived exosomes (M1‐Exos) transferring RPS18 to PAAD cells, we extracted M1 macrophage Exos before and after IRF7‐KO. Co‐cultivation with PANC‐1 cells was followed by high‐throughput sequencing, data processing and differential analysis. The results revealed five mRNAs significantly differentially expressed compared to the IRF7‐KO‐Exos group (Figure 6M). The patterns of expression for these five genes are illustrated in Figure 6N. Through the BioGRID database, we identified 373 genes interacting with RPS18, and the intersection led to a crucial mRNA: ILF3 (Figure 6O). Additionally, the expression levels of ILF3 in M1‐Exos and PAAD cells from WT‐Exos and IRF7‐KO‐Exos groups were assessed. The results indicated no significant change in ILF3 content in M1‐Exos between the two groups, suggesting that ILF3 in M1 macrophages is not regulated by IRF7/RPS18. Furthermore, compared to the WT‐Exos group, a substantial augmentation in ILF3 expression was noted in PAAD cells of the IRF7‐KO‐Exos group, demonstrating the regulatory influence of RPS18 from M1‐Exos on ILF3 in PAAD cells, with ILF3 expression increasing following IRF7‐KO in M1 macrophages (Figure 6P).

The results above indicate that M1 macrophage extracellular vesicles from the IRF7‐KO group are abundant in RPS18. These M1 macrophage extracellular vesicles, also known as M1‐Exos, can transfer RPS18 to PAAD cells and influence the expression of ILF3 in these cells.

### 2.7 IRF7 deficiency in M1 macrophages modulates RPS18/ILF3 axis via exosomal transfer, influencing lipid metabolism and PAAD cellular dynamics

**FIGURE 7:**
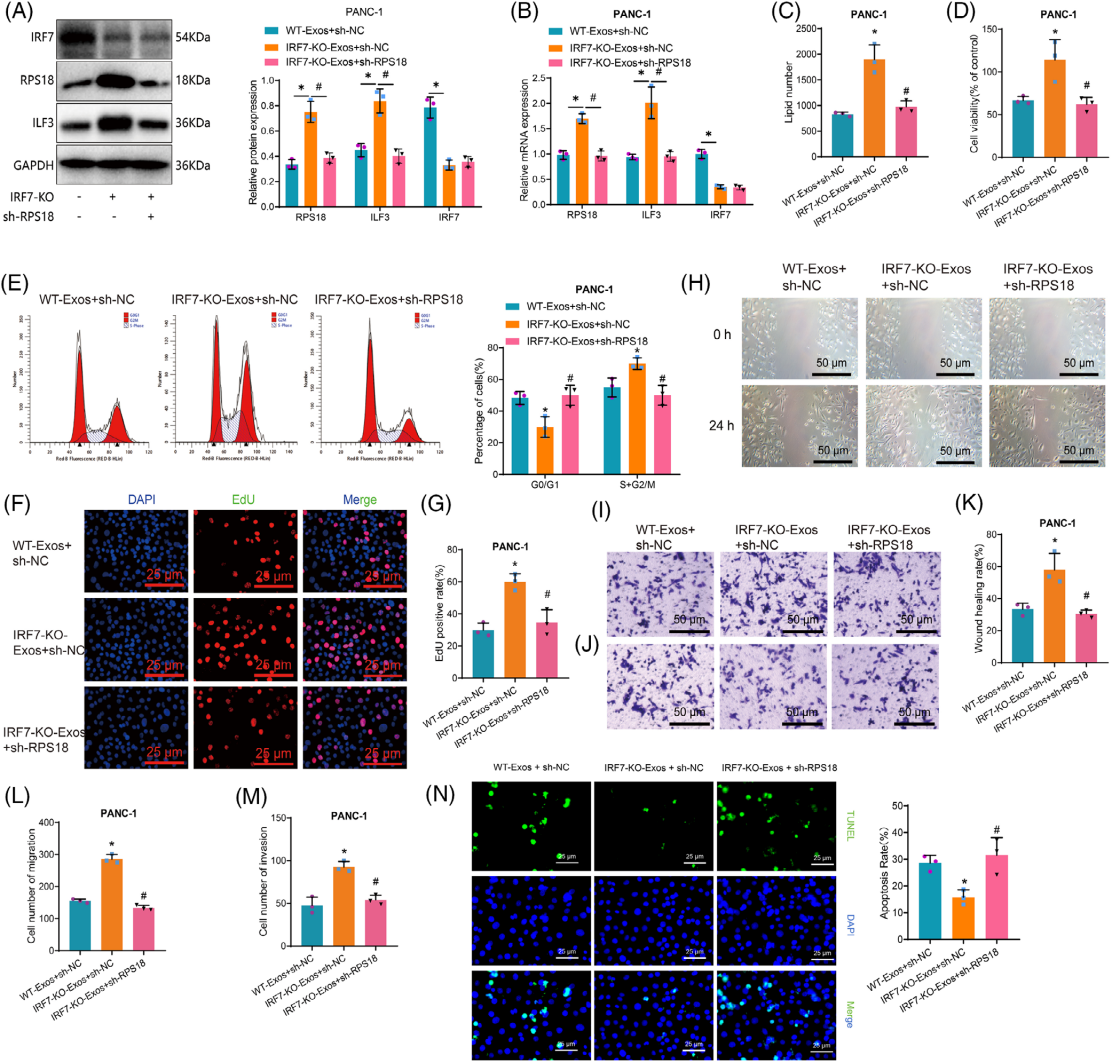
Impact of IRF7/RPS18 regulation of ILF3 expression in M1‐Exos on the biological functions of pancreatic adenocarcinoma (PAAD) cells. (A) Evaluation of protein expression levels of RPS18, IRF7 and ILF3 in PANC‐1 cells using Western blot analysis in different groups. (B) Assessment of mRNA expression levels of RPS18, IRF7 and ILF3 in PANC‐1 cells across various groups via RT‐qPCR. (C) Metabolomic analysis of total lipid content in PANC‐1 cells in each group. (D) MTT assay to measure cell viability in each group of PANC‐1 cells. (E) Flow cytometry analysis of cell cycle changes in PANC‐1 cells in each group. (F) EdU assay to assess the proliferative capability of PANC‐1 cells (scale bar: 20 µm). (G) Statistical analysis of the EdU assay results. (H) Scratch assay to assess the migration ability of PANC‐1 cells (scale bar: 50 µm). (I and J) Transwell assay to measure the migration and invasion ability of PANC‐1 cells (scale bar: 50 µm). (K) Statistical analysis of the scratch assay results. (L and M) Statistical analysis of the Transwell assay results. (N) TUNEL assay to determine the apoptosis rate of PANC‐1 cells (scale bar = 25 µm). Asterisk (*) indicates *p* < .05 compared to the WT‐Exos + sh‐NC group and symbol (#) indicates *p* < .05 compared to the IRF7‐KO‐Exos + sh‐NC group. Cell experiments were repeated three times.

Subsequent investigations have demonstrated that IRF7 suppresses the transcription of RPS18 and delivers it to PAAD cells via extracellular vesicles derived from M1 macrophages. This process effectively targets the regulation of ILF3 expression in PAAD cells. This regulation serves an essential function in governing the metabolism of PAAD lipids and various cellular functions. Initially, we validated the efficacy of three sh‐RPS18 constructs in causing the knockdown of RPS18 in PANC‐1/BxPC‐3 cell lines. The RT‐qPCR results demonstrated that sh‐RPS18‐1 exhibited the most effective knockdown. Consequently, sh‐RPS18‐1 was picked for subsequent experimental studies (Figure S9A). PANC‐1/BxPC‐3 cells were co‐cultured with M1‐Exos and RPS18 was knocked down. Results from RT‐qPCR and Western blot analysis showed significant upregulation of RPS18 and ILF3 mRNA levels and protein expression in cells treated with IRF7‐KO‐Exos + sh‐NC compared to the WT‐Exos + sh‐NC group. Conversely, IRF7 mRNA levels and protein expression were significantly reduced in the aforementioned group. Additionally, compared to the IRF7‐KO‐Exos + sh‐NC group, the IRF7‐KO‐Exos + sh‐RPS18 group exhibited significant decreases in RPS18 and ILF3 mRNA levels and protein expression in PANC‐1/BxPC‐3 cells, while the levels of IRF7 mRNA and protein expression remained unchanged (Figures 7A,B and S9B,C).

In the next step, we plan to co‐culture various types of M1 macrophages with the PAAD cell lines (PANC‐1/BxPC‐3) and assess the overall lipid metabolite levels in PAAD cells after co‐culturing using metabolomics techniques. The results demonstrated that the total lipid metabolite content in PANC‐1 and BxPC‐3 cells showed an elevation in the IRF7‐KO‐Exos + sh‐NC group in a comparison of the WT‐Exos + sh‐NC group. Conversely, when juxtaposed with the IRF7‐KO‐Exos + sh‐NC group, the total lipid metabolite content in PANC‐1 and BxPC‐3 cells was reduced in the IRF7‐KO‐Exos + sh‐RPS18 group (Figures 7C and S9D). Cell viability, proliferation, migration, invasion capabilities and cellular apoptosis were assessed using multiple assays, including MTT, flow cytometry, EdU incorporation, wound healing, Transwell migration and TUNEL staining. The findings illustrated increased cell viability, proliferation and migration ability in the IRF7‐KO‐Exos + sh‐NC group compared to the WT‐Exos + sh‐NC group. Furthermore, there was an escalation in the proportion of cells in the S+G2/M phase and a notable drop in the level of apoptosis. Conversely, the IRF7‐KO‐Exos + sh‐RPS18 group decreased cell viability, proliferation and migration ability compared to the IRF7‐KO‐Exos + sh‐NC group. Moreover, the cell population in the S+G2/M phase reduced whereas apoptotic levels rose (Figures 7D‒N and S9E‒K).

Additional evidence has been presented that supports the influence of the RPS18/ILF3 axis on pathways related to lipid metabolism and the cellular biology of PAAD. To investigate the effect, we employed gene knockdown of RPS18 and overexpression of ILF3 in PANC‐1/BxPC‐3 cells. RT‐qPCR and Western blot analysis were performed to quantify the mRNA and protein expression levels. Additionally, we evaluated the biological functions of the PANC‐1/BxPC‐3 cells. The findings demonstrated that contrasting with the sh‐NC + oe‐NC group, the expression of RPS18 and ILF3 in cells of the sh‐RPS18 + oe‐NC group exhibited a noteworthy decrease.

The study further demonstrated the impact of the RPS18/ILF3 axis on lipid metabolic pathways and the biological functions of PAAD cells. Knockdown of RPS18 or overexpression of ILF3 was performed in PANC‐1/BxPC‐3 cells. mRNA and protein expression were assessed using RT‐qPCR and Western blot, alongside the examination of biological functions in PANC‐1/BxPC‐3 cells. It was observed that in cells of the sh‐RPS18 + oe‐NC group, the expression of RPS18, ILF3, PNPLA2, CETP, LDLR and APOB significantly decreased in a comparison of the sh‐NC + oe‐NC group. Additionally, the total content of lipid metabolites significantly decreased, along with reduced cell viability, proliferation and migration abilities. A significant reduction was observed in the proportion of cells in the S+G2/M phase, while the level of cell apoptosis significantly increased. Conversely, in the sh‐RPS18 + oe‐ILF3 group relative to the sh‐RPS18 + oe‐NC group, the expression of ILF3, PNPLA2, CETP, LDLR and APOB significantly increased. RPS18 expression remained unchanged, but the total content of lipid metabolites significantly increased. Moreover, cell viability, proliferation and migration abilities notably increased, along with a surge in the proportion of cells in the S+G2/M phase and a notable decline in the level of cell apoptosis (Figure S10).

In conclusion, our findings demonstrate that the knockdown of IRF7 in M1 macrophages upregulates the transcription of RPS18 and facilitates the transfer of RPS18 to PAAD cells via M1‐Exos. As a result, this process leads to an enhanced expression of ILF3 in PAAD cells. Moreover, it promotes pathways involved in lipid metabolism and enhances the vitality, proliferation, migration and invasion of PAAD cells while blocking cellular apoptosis.

### 2.8 IRF7 modulation in M1 macrophages alters RPS18 and ILF3 expression, impacting lipid metabolism and tumour growth in an orthotopic PAAD mouse model

The in vitro experiments above confirmed that silencing IRF7 enhances RPS18 transcription and facilitates the transfer of RPS18 to PAAD cells via M1‐Exos. Moreover, this will upregulate ILF3 expression in PAAD cells, thereby bolstering lipid metabolism pathways and augmenting the vitality, proliferation, migration and invasion capability of PAAD cells while suppressing cell apoptosis. To explore whether this mechanism impacts the tumourigenic potential of PAAD cells in vivo, we developed a mouse orthotopic transplantation model of PAAD and administered M1‐Exos along with PAAD cells.

**FIGURE 8:**
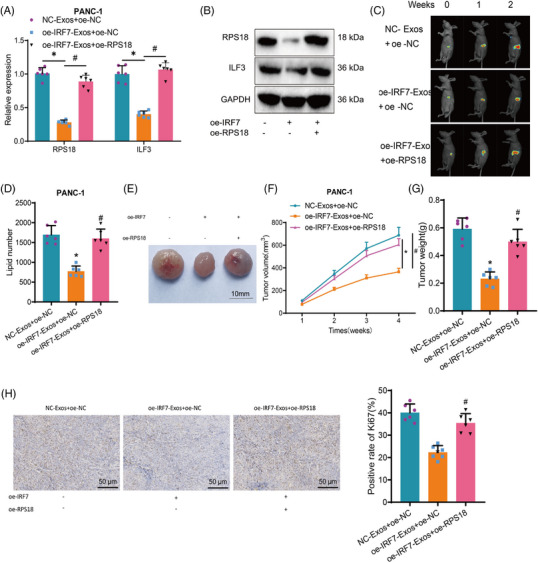
Impact of IRF7/RPS18 regulation of ILF3 expression in M1‐Exos on tumour formation in pancreatic adenocarcinoma (PAAD) cells in vivo. (A) RT‐qPCR to measure the gene expression levels of RPS18 and ILF3 in tumour tissues of each group of mice. (B) Western blot to detect the protein expression levels of RPS18 and ILF3 in tumour tissues of each group of mice. (C) Monitoring of tumour growth at different time points by bioluminescent intensity. (D) Metabolomic analysis of total lipid content in tumour tissues of each group of mice. (E) Morphology of tumour tissues in each group, with one representative example shown. (F) Tumour growth in each group of mice. (G) Tumour tissue weights in each group of mice. (H) Immunohistochemical staining to detect the protein expression levels of Ki67 in tumour tissues of each group of mice (scale bar: 50 µm). Asterisk (*) indicates *p* < .05 compared to the NC‐Exos + oe‐NC group and symbol (#) indicates *p* < .05 compared to the oe‐IRF7‐Exos + oe‐NC group. Each group consisted of six mice.

Initially, using RT‐qPCR and Western blot analysis, we observed a notable decrease in the expression of RPS18 and ILF3 in the tumour tissues of mice belonging to the oe‐IRF7‐Exos + oe‐NC group relative to the NC‐Exos + oe‐NC group. Nonetheless, RPS18 and ILF3 expression in tumour tissues of mice from the oe‐IRF7‐Exos + oe‐RPS18 group was elevated contrasting with the oe‐IRF7‐Exos + oe‐NC group (Figures 8A,B and S11A,B).

Subsequently, different groups of M1‐Exos were injected into the PAAD mouse tumour model, and tumour bioluminescence imaging was conducted at 1 and 2 weeks to monitor tumour growth. The results indicate that compared to the NC‐Exos + oe‐NC group, the growth rate of tumours in mice from the oe‐IRF7‐Exos + oe‐NC group was inhibited. In contrast, the growth rate of tumours in mice from the oe‐IRF7‐Exos + oe‐RPS18 group was even faster than that of the oe‐IRF7‐Exos + oe‐NC group (Figures 8C and S11C).

Furthermore, we compared lipid metabolism profiling results in tumour tissues from different groups of mice, along with the tumour volume and weight assessment in vivo. Our findings indicate that the lipid metabolite content in mouse tumour tissues was decreased in the oe‐IRF7‐Exos + oe‐NC group when juxtaposed with to the NC‐Exos + oe‐NC group, decreasing tumour volume and weight. Conversely, the oe‐IRF7‐Exos + oe‐RPS18 group exhibited an increase in lipid metabolite content in mouse tumour tissues in a comparison of the oe‐IRF7‐Exos + oe‐NC group, leading to an increase in tumour volume and weight (Figure 8D‒G and S11D‒G).

Ki67 expression in mouse tumour tissue was investigated through additional immunohistochemical staining. The outcomes demonstrated that the percentage of Ki67‐positive cells in the mouse tumour tissue was reduced in the oe‐IRF7‐Exos + oe‐NC group in comparison to the NC‐Exos + oe‐NC group. Conversely, the percentage of Ki67‐positive cells in the mouse tumour tissue encountered a rise in the oe‐IRF7‐Exos + oe‐RPS18 group contrasting with the oe‐IRF7‐Exos + oe‐NC group (Figures 8H and S11H). These results suggest that the overexpression of IRF7 in M1‐type macrophages leads to the inhibition of RPS18 transcription. Consequently, there is a reduction in the transfer of RPS18 to PAAD cells by M1‐Exos, resulting in the suppression of ILF3 expression in PAAD cells. Furthermore, this inhibition of ILF3 expression further impedes lipid metabolism, ultimately suppressing tumour formation in PAAD cells in vivo.

## 3 DISCUSSION

In recent years, the significance of macrophages in various tumour malignancies has been increasingly acknowledged, with particular emphasis on the interaction and regulation of M1 and M2 macrophages within the tumour microenvironment.^34^ However, the involvement of macrophages in regulating lipid metabolism and their impact on tumour progression in PC remains unclear.^35^ This study utilised high‐throughput sequencing and bioinformatics methods to uncover that IRF7 in M1 macrophages plays a crucial role in regulating PC progression. This finding contrasts with previous studies on macrophages in different types of tumours, thus emphasising the distinctive regulatory mechanisms underlying PC.^36^

IRF7 is regarded as a transcription factor in numerous diseases. However, its role and regulatory mechanisms in PAAD have been sparsely reported.^37^, ^38^ The present study discovered that IRF7 can suppress the transcription of RPS18. However, previous studies have primarily concentrated on investigating the role of IRF7 in viral infection and immune response. In contrast, our findings offer fresh insight into its function in tumour biology.^39^, ^40^, ^41^ As research on Exos continues to deepen, their significance in intercellular communication is gradually becoming acknowledged. This study provides additional confirmation that M1 macrophages are capable of transferring RPS18 to PAAD cells using extracellular vesicles, which in turn regulate the expression of ILF3. This study uncovers new functions of Exos in PAAD and complements previous research on Exos in other diseases.^14^ Furthermore, the IRF7 protein may exert a direct influence on cancer cell gene expression and subsequent cell death via the extracellular vesicle pathway. Research suggests that IRF7 may also inhibit the expression of genes that promote tumour proliferation, invasion and metastasis through direct or indirect mechanisms, such as by regulating the expression of cell cycle proteins and apoptosis inhibitors such as members of the Bcl‐2 family, thereby altering the survival and proliferation capabilities of cancer cells.^42^ Additionally, as a transcription factor, IRF7 can directly bind to gene promoter regions, activating the expression of a range of antiviral and immune response‐related genes, such as type I interferons (IFN‐α/β) and their downstream signalling pathways.^43^ Type I interferons (IFN‐α/β) can induce extensive gene expression changes, including those involved in metabolic regulation. IFN can affect cell metabolism indirectly by inhibiting glycolysis, activating oxidative phosphorylation, among other means, impacting the lipid metabolism status of tumour cells, for example, by influencing cholesterol synthesis or fatty acid oxidation.^44^

While lipid metabolism plays a crucial role in several diseases, the precise mechanisms and effects concerning PAAD remain incompletely understood. This study explored the influence of IRF7 on lipid metabolism in PAAD, offering novel insights and new avenues for metabolic research in this disease.

The CRISPR/Cas9 gene editing technology offers a rapid and efficient approach to investigating the function of select genes.^45^ Using this technique, we generated cell lines by knocking out and overexpressing key genes, which are crucial tools for future functional experiments. In comparison to traditional functional verification methods, this approach not only enhances efficiency but also boosts accuracy.

The role of IRF7 in M1 macrophages and its influence on the progression of PAAD present a novel therapeutic target. Modulating the expression of IRF7 or its downstream pathways may offer new therapeutic prospects for patients with PAAD. In comparison to conventional treatment methods, this targeted therapy has the potential to be more specific and efficient.^46^

PAAD is an extremely malignant tumour with an unfavourable prognosis.^47^ Although previous research has contributed to our understanding of the biology and treatment of (the subject), numerous unresolved questions remain.^48^, ^49^, ^50^ This study offers a novel perspective and direction, establishing a firm groundwork for future research on PAAD.

**FIGURE 9:**
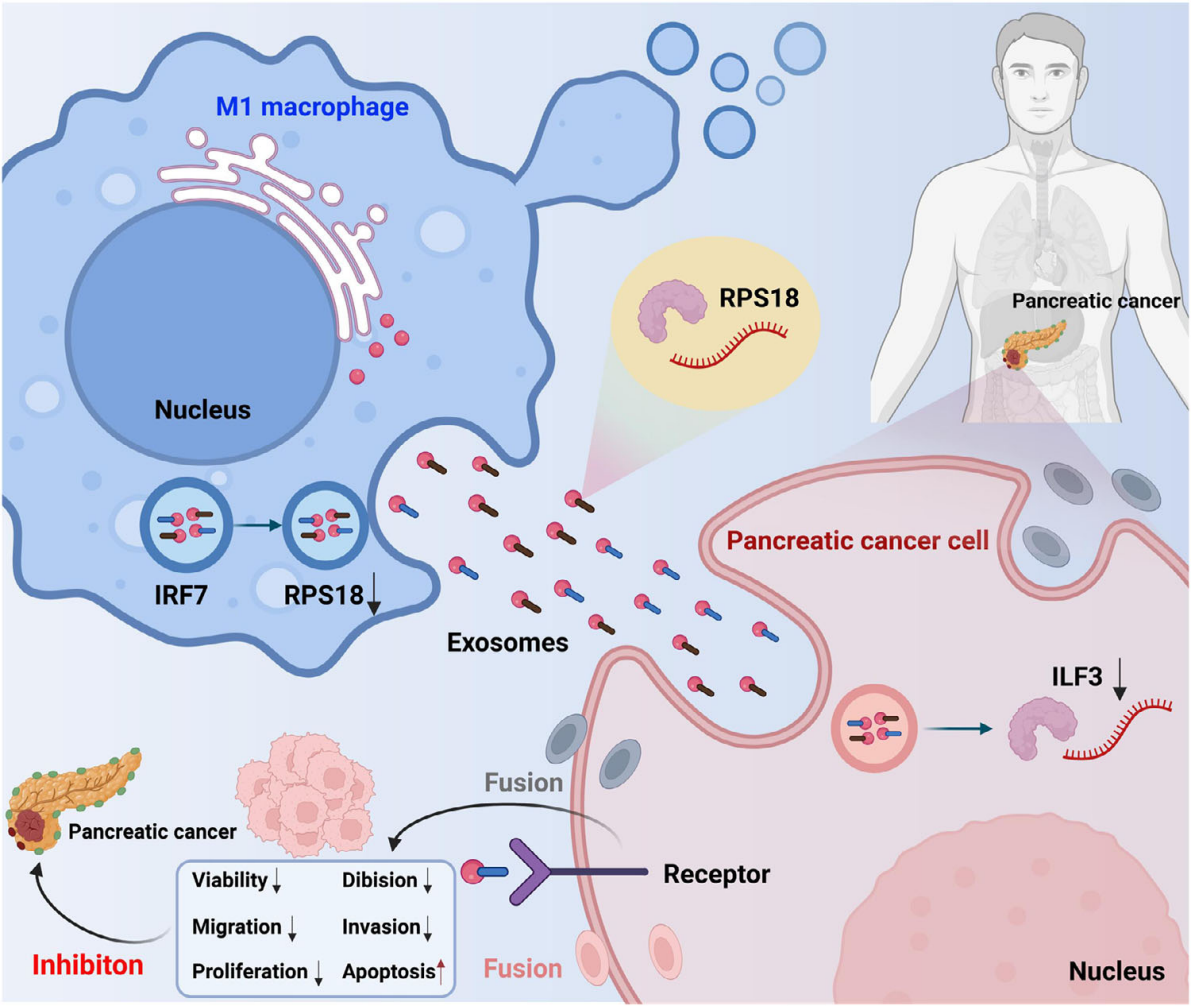
Schematic representation of the molecular mechanism by which M1 macrophages inhibit pancreatic ductal adenocarcinoma (PDAC) by transferring lipid metabolism‐related genes IRF7/RPS18.

Based on the results above, the following conclusions could be drawn preliminarily: increased expression of IRF7 in M1 macrophages possibly hampers the transcription of RPS18, diminishes the transfer of RPS18 to PAAD cells via M1‐Exos, consequently repressing the expression of ILF3 in PAAD cells. As a result, lipid metabolism pathways are inhibited, leading to suppressed activity, proliferation, migration and invasion of PAAD cells. Additionally, apoptosis is promoted, and tumour formation in PAAD cells is further suppressed (Figure 9).

This study elucidates the association between IRF7/RPS18 in M1 macrophages and lipid metabolism in PAAD cells, and their roles in proliferation, migration and apoptosis. This offers a fresh perspective on the onset, advancement and immunotherapy of PAAD. Through in‐depth analysis of PAAD tissues using scRNA‐seq and ST, specific cell populations and signalling pathways associated with cancer can be more accurately identified. If further validation confirms the function and mechanism of IRF7, it could become a potent target for PAAD therapy, providing patients with more treatment options and improved prognosis.

Despite the use of in vitro and in vivo models, the significant differences between the human body's environment and experimental conditions, as well as the limited number of scRNA‐seq samples for PAAD and the absence of female samples, may restrict the applicability of the experimental results in humans. While the CRISPR/Cas9 gene editing technology used in this study is efficient, it may also have off‐target effects, leading to unintended genetic mutations that could affect the accuracy of the experimental results. Additionally, the extraction and purification methods of Exos may impact their composition and function, as different methods could yield distinct exosome subpopulations, potentially influencing the experimental outcomes, and the uptake mechanisms of Exos in the body may be more complex, which cannot be fully simulated under experimental conditions.

The investigation centers on exploring the correlation between IRF7 and RPS18 in PAAD. However, PAAD is a complex multifactorial disease, and there may be other unknown or unstudied key factors. M1 macrophages have unique roles in many physiological and pathological processes. Studying only from the perspective of IRF7 may overlook other important mechanisms. Research lacks a thorough investigation of the impact of IRF7 on PAAD cancer cells and the differential expression of IRF7 in different PAAD cells. Future exploration could delve into the precise interaction mechanisms between IRF7 and RPS18, how M1 macrophages influence other mechanisms of PAAD cells, the differential expression of IRF7 in different PAAD cells, and the impact of IRF7/RPS18 on PAAD cells. Given the critical role of macrophages in many cancers, the findings of this study may provide insights for research and treatment of other cancers. If IRF7 indeed has a significant impact on PAAD, clinical trials could be considered in the future to determine the effectiveness of targeting IRF7 for patient treatment.

The impact of this study lies in using advanced scRNA‐seq technology to reveal that IRF7 regulates M1 macrophages by inhibiting the transcription of RPS18. Through extracellular vesicles as mediators, IRF7 functions to regulate the growth inhibition in PAAD cells, providing us with a new perspective on the correlation between PAAD cells and macrophages, potentially introducing new opportunities for averting, detecting and managing PAAD.

## 4 MATERIALS AND METHODS

### 4.1 Clinical sample collection

The study included cancer tissues from three patients who received PAAD treatment at our hospital between January and December 2022. Additionally, adjacent non‐cancerous tissues located 2 cm from the tumour site were selected as controls.^51^, ^52^ Before surgery, none of the patients received anti‐tumour treatments such as radiotherapy or chemotherapy and had no history of other diseases. Following organ excision, the sample was cryopreserved in liquid nitrogen and maintained at −80°C. All participants had perused and endorsed informed consent papers before the surgery. Additionally, the study has obtained approval from the ethics committee and strictly adheres to the Helsinki Declaration. For detailed clinical data on these three PAAD patients, please refer to Table S2.^53^, ^54^

### 4.2 Single‐cell sequencing

The pancreatic tissues obtained were washed with ice‐cold PBS to eliminate any residual tissues other than the pancreas and disrupt the tissues. Subsequently, 1 mg/mL of collagenase (C2674, Sigma‒Aldrich) was added at 37°C for 10‐min tissue digestion, followed by an additional 5‐min incubation using trypsin/Ethylenediaminetetraacetic Acid (EDTA) (25200072, Gibco) to prepare a single‐cell suspension. The C1 single‐cell automatic preparation system (Fluidigm, Inc.) was utilised to capture individual cells, lyse them within the chip post‐capture to release mRNA and perform reverse transcription to generate cDNA. The cDNA, post‐lysis and reverse transcription, underwent pre‐amplification on a microfluidic chip for ease of subsequent sequencing. The pre‐amplified cDNA was used for library construction and single‐cell sequencing on the HiSeq 4000 Illumina platform (parameters: paired‐end reads, read length of 2 × 75 bp, approximately 20 000 reads per cell).^55^

We utilised the criteria of having 200 < nFeature_RNA < 5000 and percent.mt < 25 to conduct quality control on the data. Afterwards, we selected the top 2000 genes with the greatest variability in expression.^56^

### 4.3 TSNE clustering analysis and cell annotation

PCA was applied to reduce the dimensionality of the scRNA‐seq dataset, focusing on the top 2000 genes with the highest variability. We selected the first 14 PCs for downstream analysis using the Elbowplot function from the Seurat package. We utilised Seurat's FindClusters function to identify major subpopulations of cells, with a default resolution of .2. Next, we utilised the t‐SNE algorithm for non‐linear dimensionality reduction of scRNA‐seq data. Finally, we annotated the cells using established marker genes specific to the cell lines and employed the online resource CellMarker. Please consult Table S3 for the annotated marker genes for each cell type.^57^

### 4.4 Bioinformatics analysis of public database microarray data

To obtain the ST dataset GSE211895, it was advised to download it from the GEO database (https://www.ncbi.nlm.nih.gov/gds). GSE211895 includes three cases of PAAD tissue sections from PAAD patients and their ST sequencing data. We integrated the mentioned scRNA‐seq data with the 10× Visium ST data obtained from the GSE211895 dataset employing the anchor‐based integration pipeline implemented in Seurat. The transfer of cell‐type annotations from scRNA‐seq to ST is facilitated by this technique. The results of cell‐type prediction in Seurat were imported into an R package called SPOTlight to annotate and visualise the cell types at each spatial location.^56^

The chip data and associated clinical data of the PAAD dataset GSE183795 were obtained from the GEO website (https://www.ncbi.nlm.nih.gov/geo/). The GSE183795 dataset included 139 pancreatic tumour samples, 102 adjacent non‐tumour tissue samples and three normal pancreatic samples from patients with PDAC. Expression levels of 274 DEGs in Macrophages were extracted using the ‘limma’ package in R. Subsequently, a prognostic risk scoring model was developed with the ‘caret’, ‘glmnet’, ‘survival’ and ‘survminer’ packages in R. Analysis of differential gene expression in high and low‐expressed genes in PAAD samples was conducted through Gene Ontology (GO) and KEGG pathway enrichment analysis using the SangerBox database (http://sangerbox.com/home.html). The gene set relevant to the ‘LIPID METABOLIC PROCESS’ pathway was acquired from the Genecards database (https://www.genecards.org/). Visualisation of the top 20 genes based on relevance score was performed using GraphPad software.

Immune infiltration analysis was performed on the transcriptome data for PAAD in the TCGA database, utilising the EPIC database (https://epic.gfellerlab.org/). The prediction of the binding sites of IRF7 and RPS18 was made using the LASAGNA‐Search 2.0 database, which was available at https://BioGRID‐lasagna.engr.uconn.edu/lasagna_search/.^58^

### 4.5 Development and maintenance of in vitro cellular models

The human PAAD cell lines PANC‐1 (CRL‐1469) and BxPC‐3 (CRL‐1687), as well as the human monocyte cell line THP‐1 (TIB‐202), were obtained from the American Type Culture Collection (ATCC) in the United States. The PANC‐1 cell culture was sustained in Dulbecco's Modified Eagle Medium (DMEM) medium (11965092, Gibco) enriched with 10% Fetal Bovine Serum (FBS), 10 µg/mL streptomycin and 100 U/mL penicillin. RPMI‐1640 medium (A1049101, Gibco) containing 10% FBS and 1% penicillin/streptomycin was employed for the cultivation of BxPC‐3 and THP‐1 cells. Once the density of THP‐1 monocytes reaches 1.0 × 10^5^ cells/cm^2^, a concentration of 50 nM phorbol‐12‐myristate‐13‐acetate (PMA, HY‐18739, MedChemExpress) was introduced to a cell culture chamber (CLS3412, Corning) with a pore size of .4 µm. This concentration was maintained for 48 h to trigger the differentiation process from monocytes to macrophages. After culturing, the macrophages were exposed to 100 ng/mL IFN‐γ (HY‐P7025A, MedChemExpress) for 24 h to induce their differentiation into M1‐like macrophages. It resulted in the generation of an in vitro model of M1‐like macrophages.^59^, ^60^ In investigating the role of extracellular vesicles secreted by M1 macrophages, we treated the cells with a 5 µM neutral sphingomyelinase (nSMase) inhibitor, GW4869 (HY‐19363, MedChemExpress), to suppress the secretion of extracellular vesicles by the macrophages.^61^

The 293T cell line (ATCC, CRL‐3216) was purchased and maintained in DMEM medium (Gibco, 11965092) supplemented with 10% FBS, 10 µg/mL of puromycin and 100 U/mL of penicillin. The cells mentioned above were grown in a humidified incubation chamber (Heracell Vios 160i CR CO_2_ incubator, 51033770, Thermo Scientific) at 37°C and a CO_2_ concentration of 5%. Passage culture was conducted when cells have reached 80%‒90% confluence.^62^

### 4.6 ChIP‐qPCR

The processed cells were crosslinked by treating them with 1% formaldehyde at ambient conditions for a quarter of an hour. Cell lysis was carried out using Radio Immunoprecipitation Assay (RIPA) lysis buffer (89900, Thermo Fisher) supplemented with proteinase inhibitors and nuclease to facilitate chromatin release. Chromatin disruption was achieved through ultrasonic disruption using a small‐volume instrument known as an ultrasonic cell disruptor. PEG 8000 (P8270, Solarbio) was employed and transferred to a new tube for chromatin precipitation. Chromatin immunoprecipitation was conducted by adding the rabbit anti‐human IRF7 antibody (ab238137, Abcam) to the chromatin mixture to selectively capture the specific transcription factor.

Magnetic bead precipitation was carried out using protein A/G magnetic beads (P2012, Beyotim) to precipitate the target antibody‒chromatin complexes. Washing: a series of washing buffers with different concentrations and salt levels were employed to cleanse the protein A/G magnetic beads, removing non‐specific antibody‒chromatin complexes and impurities. The mixture was heated at 65°C for .5 h to denature the crosslink, eliminating the protein‒DNA crosslinks. The releasing buffer consisted of 5 M NaCl, 20 mg/mL proteinase K and 10% SDS. Reverse transcription was performed using the PrimeScript RT Master Mix (RR036A, Takara) to convert DNA into cDNA, which was subsequently detected by PCR.

qPCR detection was conducted by using either SYBR Green Master Mix (RR420A, Takara) or qPCR reagent kits from alternative suppliers to detect fluorescence signals emitted by the reaction system in the PCR machine. Amplification and detection were then performed using the QuantStudio 6 and 7 Flex Real‐Time PCR systems. Primer sequences for RPS18 were designed using the Primer‐BLAST online tool. Table S4 contains the primer sequences. Analysis of gene expression levels was carried out employing the 2^−ΔΔCt^ formula, and the enrichment level of the RPS18 gene was determined.^63^

### 4.7 Flow cytometry

Staining of differentiated THP‐1 monocyte‐derived macrophages was conducted through the subsequent antibodies: APC‐conjugated anti‐CD86 (17‐0869‐42, Thermo Fisher), FITC‐conjugated anti‐CD163 (MA5‐17719, Thermo Fisher) and PE‐conjugated anti‐IRF7 antibody (12‐5375‐42, Thermo Fisher). Sort IRF7+ into M1 or M2 cells. After thorough mixing, incubate at 4°C in the absence of light for 30 min. Add 2 mL of PBS solution (P4417, Sigma‒Aldrich), centrifuge at 4°C and 1500 *g* for 10 min, and then discard the supernatant. The sample was fixed by adding more than 2% formaldehyde (30525‐89‐4, Sigma‒Aldrich) to a PBS solution and then placed in the dark at 4°C for 24 h. Subsequently, an analysis of the sample was conducted using the FACS Aria II flow cytometer manufactured by BD Bioscience in the United States.^64^

The cell handling and processing methods remain consistent with the procedures above when utilising flow cytometry for the analysis of cell cycle progression. The test cells were immersed in 70% ethanol at 4°C for an extended period. Following centrifugation at 800 *g*, the supernatant was removed. Subsequently, cell incubation took place at 37°C within a binding buffer solution incorporating 10 µg/mL of RNase A, lasting for 30 min. Furthermore, the cells were treated with 50 µL of propidium iodide (PI, 50 mg/L; 40710ES03, YEASEN) and incubated for half an hour. Flow cytometry analysis was finally conducted using BD BioPAADence and BD LSRFortessa to evaluate cellular progression through the cell cycle.^65^

### 4.8 CRISPR/Cas9

IRF7‐KO cells were created using CRISPR/Cas9 technology. The specific guide RNA sequences used were as follows: IRF7‐sgRNA1: 5′‐CAAGGAGTTCGAGCTCAGCC‐3′ (PAM: NGG) and IRF7‐sgRNA2: 5′‐CAAGCTGTATGCTCTCAGCC‐3′ (PAM: NGG). The sgRNA was inserted into the Lenti‐CRISPR v2 vector containing the *Staphylococcus aureus* Cas9 nuclease gene (Haneng Biological Technology [Shanghai] Co., Ltd.). IRF7‐KO cells were generated by transducing cells with the lentiviral Lenti‐CRISPR v2 vector and utilising the CRISPR/Cas9 editing system. Transfected cells carrying sgRNA plasmids and donor sequences were selected using a concentration of 4 µg/mL puromycin (A1113803, Gibco). IRF7‐KO cells were obtained by restricting dilution cloning and screening for viable cells. It was followed by identifying IRF7‐KO cells using RT‐qPCR and Western blot. Finally, DNA sequencing was employed to confirm the presence of IRF7‐KO cells.^66^, ^67^

The M1‐Exos groups were divided into two categories: (1) IRF7‐WT group, which refers to the wild‐type group without having IRF7 knocked out, and (2) IRF7‐KO group, which refers to the group with IRF7 knocked out. After 48‐h transfection, the Exos derived from M1 macrophages before and after knockout were separated and named WT‐Exos and IRF7‐KO‐Exos, respectively. They were then stored at −80°C for future use.

### 4.9 Lentiviral vector construction

Analysis of potential short hairpin RNA target sequences based on GeneBank for human cDNA sequences. Three diverse sequences targeting RPS18 were initially formulated, with a sequence devoid of interfering factors as a negative control (sh‐NC). The primer sequences can be found in Table S5. GenePharma synthesised these oligonucleotides. A lentiviral vector tailored for gene suppression, pLKO.1, allows for the creation of a lentiviral packaging mechanism. The packaged virus and the target vector were co‐transfected into 293T cells using lipofectamine 2000 at 80%‒90% cell confluence. Subsequent to a 2‐day cell culture period, the supernatant containing virus particles was collected through filtration and centrifugation. The collection of samples from the growth site and subsequent testing of virus titres. Genechem constructed and packaged the lentivirus for gene overexpression. The lentiviral gene overexpression vector used was LV‐PDGFRA.^68^, ^69^, ^70^, ^71^

### 4.10 Cell transfection

By employing trypsin digestion followed by trituration, a cell suspension containing 5 × 10^4^ cells/mL was derived from cells in the logarithmic growth stage. The inoculation suspension was distributed into a six‐well plate, adding 2 mL to each well. Before constructing the in vitro cell model, various lentiviruses (Multiplicity of Infection (MOI) = 10, virus titre of 1 × 10^8^ TU/mL) were applied to the culture medium of cells and incubated for 48 h. Stable cell lines were screened using 2 µg/mL puromycin (UCOE03, Sigma‒Aldrich) for 2 weeks.

The cell transfection groups were classified as follows: (1) sh‐NC group, which was transfected with lentivirus constructed with a negative control vector; (2) sh‐RPS18 group, which was transfected with lentivirus constructed with a sh‐RPS18 vector; (3) oe‐NC group, which was transfected with lentivirus constructed with an oe‐NC vector; (4) oe‐IRF7 group, which was transfected with lentivirus constructed with an oe‐IRF7 vector; (5) oe‐RPS18 group, which was transfected with lentivirus constructed with an oe‐RPS18 vector; and (6) oe‐ILF3 group, which was transfected with lentivirus constructed with an oe‐ILF3 vector. RNA and protein levels were examined at 36 and 48 h to validate the knockdown efficiency after 48 h of transfection. Plasmids previously alluded to underwent a design and synthesis process by Guangzhou Rebo Biological Technology Co., Ltd.^72^

### 4.11 RT‐qPCR

The comparative levels of gene expression were assessed through the utilisation of Trizol reagent (15596026, Invitrogen) for the isolation of total RNA from cellular structures or tissue specimens. The concentration and purity of the total RNA were measured at 260/280 nm using NanoDrop LITE (ND‐LITE‐PR, Thermo Scientific). Total RNA was extracted and reverse transcribed into cDNA using the PrimeScript RT reagent Kit with gDNA Eraser (RR047Q, TaKaRa). Furthermore, the RT‐qPCR detection of each gene was conducted employing the SYBR Green PCR Master Mix reagents (4364344, Applied Biosystems) and the ABI PRISM 7500 Sequence Detection System (Applied Biosystems).

The primers for each gene were synthesised by the TaKaRa company (Table S6). The reference gene employed for standardisation was GAPDH. We analysed the relative expression levels of each gene via the 2^−ΔΔCt^ approach. The ΔΔCt calculation involved subtracting the mean Ct value of the target gene in the experimental group from the mean Ct value of the reference gene in the same group, followed by subtracting the mean Ct value of the target gene in the control group from the mean Ct value of the reference gene in the control group. The RT‐qPCR examinations were carried out three times.^73^, ^74^, ^75^

### 4.12 Western blot

The tissues or cells were initially gathered and an improved RIPA lysis buffer (P0013B, Beyotime Biotechnology Co., Ltd.) supplemented with a proteinase inhibitor was used for lysis. Subsequently, the protein concentration was measured using the Bicinchoninic Acid (BCA) protein quantification kit (P0012, Beyotime Biotechnology Co., Ltd.). Protein separation was carried out employing 10% Sodium Dodecyl Sulfate Polyacrylamide Gel Electrophoresis (SDS‒PAGE), followed by transfer to a Polyvinylidene Fluoride (PVDF) membrane. To prevent nonspecific binding, the solution was supplemented with 5% Bovine Serum Albumin (BSA) and left at room temperature for 2 h for blocking. The diluted primary antibody was separately added and incubated at room temperature for 1 h. All antibodies used in the study were rabbit anti‐human. For further details, refer to Table S7. After washing, the sample was incubated with either Horseradish Peroxidase (HRP)‐conjugated sheep anti‐rabbit secondary antibody (ab6721, 1:2000, Abcam) or sheep anti‐mouse secondary antibody (ab6785, 1:1000, Abcam) at room temperature for 1 h. Equal volumes of solutions A and B from the Pierce ECL Western blot substrate (32209, Thermo Scientific) were taken and thoroughly mixed in a darkroom. This mixture was then applied to the membrane. Subsequently, the membrane was placed into a gel imaging system for exposure and imaging. The images were captured using the Bio‐Rad imaging system (Bio‐Rad), and the grayscale quantification of the bands in the Western blot images was performed using ImageJ software. GAPDH was used as the internal reference. The experiment was repeated three times each.^74^

### 4.13 Co‐culture of cells

After isolating extracellular vesicles derived from M1‐type macrophages, these vesicles were separately co‐cultured with PANC‐1/BxPC‐3 cells for 36 h. Subsequently, the PAAD cells were harvested for further experiments.^76^, ^77^, ^78^, ^79^

The cell co‐culture groups were as follows: (1) IRF7‐WT group—co‐culture of extracellular vesicles from wild‐type M1 macrophages with PANC‐1/BxPC‐3 cells; (2) IRF7‐KO group—co‐culture of extracellular vesicles from IRF7‐KO M1 macrophages with PANC‐1/BxPC‐3 cells; (3) oe‐NC group—co‐culture of extracellular vesicles from M1 macrophages transfected with empty plasmid with PANC‐1/BxPC‐3 cells; and (4) oe‐IRF7 group—co‐culture of extracellular vesicles from M1 macrophages transfected with IRF7 plasmid with PANC‐1/BxPC‐3 cells.

### 4.14 mRNA stability assay

PANC‐1/BxPC‐3 cells were cultured overnight in a 12‐well plate. After 48 h of pretreatment, RNA transcription was inhibited using 5 µg/mL actinomycin D (Act‐D, Sigma, A9415). Cells were collected at 0, 3 and 6 h post‐Act‐D addition. The mRNA expression levels at the specified time points were quantified for each group.^80^

### 4.15 Extraction of lipid metabolites in organisms and cells

#### 4.15.1 Metabolite extraction

PAAD cells were co‐cultured and seeded within cell culture dishes. Cells reaching confluence were digested with trypsin–EDTA (25200072, Gibco) and subsequently rinsed with a PBS buffer. The washed cells or pretreated PAAD tissues were subjected to freeze‒drying. Afterwards, the freeze‐dried samples were added 100 µL of a pre‐cooled ethanol/acetonitrile/water solution (2:2:1, v/v/v). The mixture was vortexed and underwent low‐temperature ultrasound treatment for 30 min, followed by freezing at −20°C for 10 min. Subsequently, the mixture was centrifuged at 4°C and 14 000 *g* for 20 min. After additional vacuum drying, 100 µL of a 1:1 acetone/water solution (v/v) was added to the supernatant. After vortexing, the mixture underwent centrifugation at 4°C and 14 000 *g* for 15 min. The resulting supernatant was subsequently employed for analysis.

#### 4.15.2 HPLC conditions

The sample was separated using an ultra‐high‐performance liquid chromatography HILIC column. At a constant chromatographic temperature of 40°C, the flow rate was maintained at .4 mL/min, with an injection volume of 2 µL. Mobile phase A comprises an aqueous solution containing 25 mmol/L of ammonium acetate and 25 mmol/L of ammonia, whereas mobile phase B consists of acetonitrile. In the gradient elution mode, the initial condition was 95% B, held for 0‒.5 min. Within 7 min, the B composition linearly decreases from 95% to 65%. Subsequently, over the next 8 min, B linearly decreases from 65% to 40% and remains constant at 40% for 8−9 min. Finally, within the next 9 min, B linearly increased from 40% to 95% and was held constant at 95% for 9−12 min.

The Q‐TOF mass spectrometer was operated under the following conditions: the method for ESI source settings involved setting ion source gas I and ion source gas II to 50, shielding gas to 30, and maintaining a source temperature of 500°C. The ion spray voltage for both negative and positive ion modes was ±5500 V. Generate product ion scans utilising information‐dependent acquisition under high sensitivity mode. The collision energy was set at 35 ± 15 eV, and the de‐clustering potential in both the negative and positive ion modes was ±80 VV.^81^, ^82^, ^83^

### 4.16 Quantification of lipid metabolites by liquid chromatography‒mass spectrometry

The raw mass spectrometry data, specifically the whiff. Conversion of the scan file to the .mzXML format was accomplished using ProteoWizard MSConvert. Peak alignment, retention time correction and peak area extraction were executed with XCMS. The Collection of Algorithms for Metabolite Profile Annotation (CAMERA) software annotates isotopes and adducts in metabolite profiles. Compound identification of all lipid and other metabolites was achieved by comparing their *m*/*z* (<10‰) and MS/MS spectra with those in the mzCloud (www.mzcloud.org/), mzVault and Masslist databases.^81^, ^84^

### 4.17 MTT

The test cells were seeded onto a 96‐well cell culture plate at a density ranging from 3 × 10^4^ to 5 × 10^4^ cells/mL and then incubated for 48 h. MTT solution (10 mg/mL, ST316, Beyotime Biotechnology Co., Ltd.) was added to the cell suspension and incubated for 4 h. Afterwards, dimethyl sulfoxide was added and shaken for 10 min. The absorbance was measured at 490 nm using a spectrophotometer (Laspec).^85^

### 4.18 EdU experiment

The test cells were inoculated into a 24‐well plate, and EdU (C10310‐2, Guangzhou RiboBio Co., Ltd.) was added to the culture medium to achieve a 10 µmol/L concentration. The cells were then incubated in a culture chamber for 2 h. Upon removal of the culture medium, the cells were treated with a 4% paraformaldehyde solution in PBS and left at room temperature for 15 min for fixation. Following fixation, the cells were rinsed twice with 3% BSA in PBS, then exposed to PBS containing .5% Triton‐100 at room temperature for 20 min. Subsequently, they underwent two additional washes with 3% BSA in PBS. Then, 100 µL of staining solution was added to each well, and the plate was incubated at room temperature in the dark for 30 min. 4′,6‐diamidino‐2‐phenylindole (DAPI) staining was performed for 5 min after mounting. Utilising a fluorescence microscope (BX63, Olympus), random observation was conducted across 6−10 fields of view, and the count of positive cells in each field was documented. The determination of the EdU labelling rate entailed dividing the quantity of positively labelled cells by the total count of positively and negatively labelled cells, then multiplying the outcome by 100%.^86^ The experiment was iterated three times in succession.

### 4.19 Scratch assay

The processed test cells were plated in a six‐well plate with a cell density ranging from 70% to 90%. Drag the pipette tip containing 200 µL across the well plate to create a visible scratch. Rinse the cells to eliminate any detached cells and introduce a fresh culture medium. The samples were placed in an environment with 5% CO_2_ concentration at a temperature of 37°C for incubation. The initial condition of the scratches was recorded at 0 h. Subsequently, observations and image capture were carried out using an inverted microscope (CKX53, Olympus). After 24 h, reevaluate the state of the scratches using an inverted microscope for observation and photography. The width of scratches was measured utilising image processing software, such as ImageJ, to calculate the cell migration distance.^87^

### 4.20 Transwell experiment

The addition of the ECM gel (E1270, Sigma‒Aldrich) was required in the upper compartment of a 24‐well Transwell plate, which has 8 µm pores. The plate was placed in a 37°C incubator for 30 min to allow the gel to solidify. After removing the cells transfected for 48 h, resuspend 10^5^ cells in a serum‐free culture medium and seed them into the upper chamber. A volume of 200 µL of cell suspension, comprising 2 × 10^4^ cells, was introduced into each well, followed by seeding into the lower chamber of the Transwell plate. Supplement the lower chamber, which contains 20% FBS, with 800 µL of culture medium. Following a 24‐h incubation at 37°C, the Transwell plate was removed, and two PBS washes were performed. Next, submerge the specimen in formaldehyde for 10 min and rinse it thrice with water. After staining with .1% crystal violet, allow it to incubate at ambient temperature for 30 min, followed by two rinses with PBS. Wipe away the cells on the surface using a cotton ball. Stained invading cells were captured using an inverted microscope (CKX53, Olympus). The invasive ability of cancer cells was then counted and analysed using ImageJ software. Transwell plates could be used without ECM gel coating for migration experiments while following the same steps as in invasion experiments.^88^, ^89^

### 4.21 TUNEL staining

The tested samples from each group were fixed with 4% paraformaldehyde (60536ES60, Yeasen Biotechnology [Shanghai] Co., Ltd.) at ambient temperature for 15 min. Subsequently, they were permeabilised with .25% Triton X‐100 at room temperature for 20 min. The cells were cultured using 5% bovine serum albumin (BSA; 36101ES25, Yeasen Biotechnology [Shanghai] Co., Ltd.) and subsequently stained with TUNEL reagent (C1086, Beyotime Biotechnology Co., Ltd.). Afterward, the slides were restained in a dark environment with DAPI staining solution (C1002, Beyotime Biotechnology Co., Ltd.). Apoptotic cell images were acquired utilising a Carl Zeiss AG confocal microscope (model: LSM 880) from Germany. Cells that show green fluorescence indicating TUNEL positivity, represent apoptotic cells. DAPI stains the nucleus, while blue fluorescence indicates the total cell count. Each group chooses five diverse perspectives to calculate the cellular apoptosis rate. The rate was obtained by dividing the number of apoptotic cells by the total cell count and subsequently multiplying by 100%.^90^

### 4.22 Luciferase assay

Utilising the Dual‐Luciferase Reporter Assay System (Promega, E1910), the fluorescence enzyme assay was executed. To validate the binding between IRF7 and the RPS18 promoter, HEK293T cells (Procell, CL‐0005) were seeded in a 48‐well plate and co‐transfected with the pcDNA3.1‐IRF7 plasmid and either the pGL3‐Basic plasmid, or the reporter gene plasmid pGL3‐RPS18 containing the approximately 1 kbp RPS18 promoter region, or the mutated form of the reporter gene plasmid pGL3‐RPS18 (all plasmids synthesised by Hanheng Biotech). The fluorescence enzyme activity was normalised using the pRL‐TK vector (Promega, LM1568). Upon completion of 48 h post‐transfection, cell lysis was conducted, and luciferase activity was assessed.^91^

### 4.23 Exosomes isolation and purification

After pretreating the M1 macrophages, collect the culture medium and remove dead cells through three rounds of low‐speed differential centrifugation. Finally, filter the medium employing a .45 µm filter membrane. The filtered medium was layered onto a 2 mL 60% sucrose cushion buffer. It should then be centrifuged at 100 000 *g* for 90 min at 4°C. Collect approximately 3 mL of the interface between the sucrose pad buffer and the culture medium. Next, carefully transfer the 10 mL interface obtained from the initial high‐speed centrifugation onto .75 mL of 60% sucrose cushion buffer. Subsequently, the mixture was centrifuged at 110 000 *g* using an SW41Ti rotor at 4°C for 12 h. Collect approximately 1 mL of the interface from each tube during the second round of high‐speed centrifugation. The prepacked qEV10 SEC column was connected to the NGC Quest 10 chromatography system. Before elution, it was necessary to equilibrate and wash the samples with .22 µm filtered and degassed PBS in 75 portions, each with a volume of 2 mL. The process was performed at a temperature of 4°C. Utilise the integrated ChromLab software to acquire the absorbance reading at a wavelength of 280 nm. The 8−14 fraction, enriched with extracellular vesicles, was combined and passed through a .45 µm membrane. It was then loaded into an Amicon Ultra‐15 centrifugal filter unit and centrifuged at 3260 *g* for 4°C to achieve further concentration.^92^

### 4.24 Identification of extracellular vesicles

Western blot analysis was conducted to identify the presence of exosome marker proteins, including CD9, CD63, CD81 and calnexin. Please refer to the earlier described Western blot section for specific operating procedures and information about the antibodies used.^94^ Every experiment underwent replication thrice to ensure robustness.

The concentration and size distribution of extracellular vesicles were determined using either the NanoSight NS300 nanoparticle tracking analyser (Malvern) or the ZetaView nanoparticle tracking analyser (Particle Metrix). Inject .5 mL of extracellular vesicles into the designated sample chamber, capture three videos for each sample, and analyse the recordings within each batch that share similar detection thresholds following the suggested protocol.^92^ The concentration of Exos was determined by taking the average of three measurements.

After ultra‐speed centrifugation, the Exos suspensions were precipitated and fixed in a solution containing 2% paraformaldehyde and 2.5% glutaraldehyde at 4°C for 1 h. The specimens were washed three times with PBS for 15 min each, fixed with 1% osmic acid for 1.5 h, and washed again three times with PBS for 15 min each. Gradient ethanol dehydration was performed, followed by overnight infiltration and embedding in epoxy resin. Polymerisation was carried out at 35°C, 45°C and 60°C for 24 h. The procedure involved cutting ultrathin sections, staining them with lead uranyl, and then observing them via a transmission electron microscope (JEM‐1011; JEOL) under an acceleration voltage of 80 kV. Images were captured using a side‐mounted Camera‐Megaview III (Soft Imaging System).^33^ Repetition of each experiment was conducted three times to validate the results.

### 4.25 Observation of cellular uptake of Exos by immunofluorescence staining

The M1‐type macrophage‐derived Exos was inoculated into a 24‐well plate, and Dil dye was added to 40 μg Exos (C1036, Beyotime Biotechnology Co., Ltd, Shanghai) to achieve a final concentration of 25 μM. It then reacted at room temperature for 30 minutes to remove the unbound dye by ultracentrifugation. Subsequently, rinse the cells three times with PBS and then fix them with 4% polyformaldehyde (AR1068, BOSTER Biological Technology Co., Ltd.) for a duration of 30 min. The cell nuclei were finally stained with 4′,6‐diamidino‐2‐phenylindole (DAPI, C1005, Beyotime Biotechnology Co., Ltd.) for 30 min. Subsequently, the cells were photographed using a BX53 fluorescence microscope (Olympus) equipped with a camera at ×400 magnification. Process the images using ImageJ Pro Plus 6.0 software for analysis.^95^, ^96^

### 4.26 High throughput sequencing and analysis of PAAD cells cocultured with extracellular vesicles

Extracellular vesicles were isolated from M1 macrophages from the IRF7‐WT group (*N* = 3) and the IRF7‐KO group (*N* = 3). Subsequently, the two groups of extracellular vesicles were co‐cultured with PANC‐1 cells. Total RNA was extracted from 6 samples utilising the Total RNA Extraction Kit (12183555, Invitrogen), and the OD value of the resulting total RNA was quantified. Assess the integrity of these total RNAs through agarose gel electrophoresis. High‐quality total RNA was first reverse transcribed to generate cDNA, which was then used for RNA library construction. Utilising Illumina's NextSeq 500, sequencing was conducted. The raw image data acquired from sequencing was converted into raw reads by performing base calling. Quality assurance of the raw reads was ensured by utilising the cutadapt tool to remove sequencing adapter sequences and filter low‐quality sequences. The reads that passed the quality filtering step were referred to as ‘clean reads’. Alignment to the human reference genome was performed using the Hisat2 software for the sequences. After alignment, gene expression was quantified through the R software package to generate a gene expression matrix.^97^

The R package ‘limma’ filters DEGs in high‐throughput sequencing data. The criteria for filtering were |log_2_ FC| > 1 and adj. *p*‐value < .01. The ggplot2 R package plots volcano plots, while the pheatmap R package plots heatmaps. Draw Venn diagrams using the Xiantao Academic Database (https://www.xiantaozi.com/). The BioGRID database (http://had.co.nz/ggplot2/) was used to predict genes interacting with RPS18.^98^

### 4.27 In vivo animal experiments

Mice of the C57BL/6J strain, male gender, aged 6−8 weeks and weighing 25−30 g, were sourced from our Experimental Animal Research Center. The mice were accommodated and raised in a SPF animal laboratory. The humidity in the laboratory was maintained between 60% and 65%, while the temperature was controlled at 22°C−25°C. The mice were provided sufficient food and water under alternating light and darkness every 12 h. The experimental trials commenced one week after the adaptive feeding period, and the mice's health condition was assessed before the onset of the experiment. Adherence to the institute's ‘Guidelines for the Care and Use of Laboratory Animals’ was paramount in all animal research activities.^99^

Subcutaneously inject 5 × 10^5^ PANC‐1/BxPC‐3 cells into mice, allowing them to grow in vivo for 10 days. Since this cell line was derived from male mice, exclusively male mice were selected for this study to mitigate immune rejection and ensure uniform tumour growth. The subcutaneous tumour was surgically removed under general anesthesia using a posterior subcostal incision. Subsequently, it was dissected into tumour fragments measuring approximately 1 mm in diameter. The tumour fragment was transplanted onto the mouse's tail under general anaesthesia. Inhalation of volatile isoflurane was used again at a concentration of 2% and an oxygen flow rate of 3 L/min. Administer a dose of 1 mg/kg of pethidine to the mice before conducting the transplant procedure. The orthotopically transplanted PDAC tumour exhibited growth over 8 days, reaching an approximate diameter of 5 mm.^100^ The CRi Maestro in vivo imaging system (CRi Inc.) was utilised to analyse the bioluminescent signals emitted by PANC‐1/BxPC‐3 cells.^101^

M1‐Exos were injected intratumourally or intravenously into mice. Following the surgery, each group received an intravenous injection of 200 µg Exos originating from M1 macrophages (200 µg/100 µL PBS) thrice weekly. After 12 days of injection, anesthesia was administered to the mice prior to scanning with the CRi Maestro in vivo imaging system (Cambridge Research & Instrumentation). To image excised tissues, we induced deep anesthesia by administering isoflurane (Revode R510‐22‐10, Shenzhen), following which the mice were euthanised using cervical dislocation. Following the euthanasia of the mice, the tumours was separated and either applied to FFPE or snap‐frozen for subsequent experimental analysis.^102^

Random allocation resulted in six groups, each consisting of six mice. These groups included the following: NC‐Exos + oe‐NC group, which was co‐cultured with Exos derived from oe‐NC transfected M1 macrophages and oe‐NC transfected PANC‐1/BxPC‐3 cells; oe‐IRF7‐Exos + oe‐NC group, co‐cultured with Exos derived from oe‐IRF7 transfected M1 macrophages and oe‐NC transfected PANC‐1/BxPC‐3 cells; and oe‐IRF7‐Exos + oe‐RPS18 group, co‐cultured with Exos derived from oe‐IRF7 transfected M1 macrophages and oe‐RPS18 transfected PANC‐1/BxPC‐3 cells. Each group of mice was transplanted with PANC‐1/BxPC‐3 cells.

### 4.28 Histological staining

Haematoxylin and eosin (H&E) staining involves obtaining the tissue sample for analysis and performing fixation. After slicing the sample, the wax block was removed from the cut sections. Subsequently, the sample was deparaffinised in xylene and dehydrated in a series of ethanol concentrations: 100%, 95% and 70%. Finally, the sample was either embedded or rinsed with water. The prepared slices were immersed in the SU‐MU colouring solution (H8070 from Solarbio) and allowed to dye at room temperature for 5−10 min. After washing the slices with distilled water, they were dehydrated in 95% ethanol and stained with eosin (Solarbio, G1100) for 5−10 min. Routine dehydration, clearing and mounting procedures were followed.^103^

### 4.29 Immunohistochemical staining

Tumour tissue from mice was transplanted subcutaneously. It was then fixed overnight with 4% paraformaldehyde. Subsequently, paraffin sectioning was performed with 4 µm thickness. Deparaffinisation of the sections was carried out with xylene, followed by a gradual alcohol gradient for hydration (comprising anhydrous ethanol, 95% ethanol and 75% ethanol, with a duration of 3 min for each). To recover the antigen, the sample needs to be boiled in a .01 M citrate buffer for 15−20 min. Subsequently, it was incubated at room temperature in a 3% H_2_O_2_ solution for 30 min to deactivate the endogenous peroxidase. Afterward, the sample was treated with goat serum‐blocking solution and left at room temperature for 20 min before the excess liquid was removed. The primary antibody, Ki67 (ab16667, 1:200, Abcam), was added and incubated at room temperature for 1 h, followed by washing with PBS. Then, the secondary antibody, IgG (ab6785, 1:1000, Abcam), was added and incubated at 37°C for 20 min. After washing with PBS, the sample was incubated with streptavidin‒peroxidase (SP) at 37°C for 30 min. Subsequently, the specimen underwent cleansing using PBS followed by the introduction of DAB (P0202, Beyotime Biotechnology Co., Ltd.) to induce colour formation within a duration of 5‒10 min. Termination of the reaction was achieved through a 10‐min wash with water. The tissue segments were stained with Sudan Black B (C0107, Beyotime Biotechnology Co., Ltd.) for 2 min. Differentiation was conducted using hydrochloric acid alcohol and a water wash for 10 min. Dehydration was achieved by treating the sections with gradient alcohol until transparent, followed by xylene. Finally, two to three drops of neutral resin were added for mounting. Cell observation and counting were performed using a light microscope. Randomly selected were five high‐power fields of view from every slide. One hundred cells were observed per field of view, and the positive rate of Ki67 cells was calculated.^104^

### 4.30 Statistical analysis

In this research, analysis was conducted using R language version 4.2.1. The compilation of R language code was facilitated by RStudio integrated development environment version 2022.12.0‐353. For file management, Perl version 5.30.0 was employed. Additionally, GraphPad Prism software version 8.0 was utilised. Quantitative data are presented as mean ± standard deviation. The independent samples *t*‐test was employed for comparing two sets of data.^105^

A one‐way analysis of variance was applied for comparing data between different groups, while a two‐way analysis of variance was used for analysing data variations within groups across various time points. Bonferroni was employed for post hoc testing. The significance threshold was *p* < .05.^106^

## AUTHOR CONTRIBUTIONS

Ting Zhan, Yanli Zou, Xia Tian and Xiaodong Huang conceived and designed research. Ting Zhan, Yanli Zou, Zheng Han and Xiaorong Tian performed experiments. Weijie Liu, Mengge Chen, Mingtao Chen and Jiaxi Liu interpreted results of experiments. Ting Zhan, Yanli Zou, Weijie Liu, Xiulin Yang and Qingxi Zhu analysed data. Weijie Liu, Meng Liu and Wei Chen prepared figures. Ting Zhan, Yanli Zou, Zheng Han and Jie Tan drafted paper. Xiaodong Huang and Xia Tian edited and revised manuscript. All authors read and approved final version of manuscript.

## CONFLICT OF INTEREST STATEMENT

The authors declare they have no conflicts of interest.

## ETHICS STATEMENT

All experiments involving mice were approved by the Animal Ethics Committee of Wuhan University (No. WQ20210145).

## Supporting information

Supporting Information

## Data Availability

The data that support the findings of this study are openly available in figshare (https://doi.org/10.6084/m9.figshare.26310844).
