# Supplementary material for: Single‐cell sequencing combined with spatial transcriptomics reveals that the IRF7 gene in M1 macrophages inhibits the occurrence of pancreatic cancer by regulating lipid metabolism‐related mechanisms
Source: Clin Transl Med. 2024 Aug 8;14(8):e1799. doi: 10.1002/ctm2.1799 (PMC11310283; doi:10.1002/ctm2.1799)
Supplement: Supplementary file 1 — Supporting Information [file CTM2-14-e1799-s001.docx]

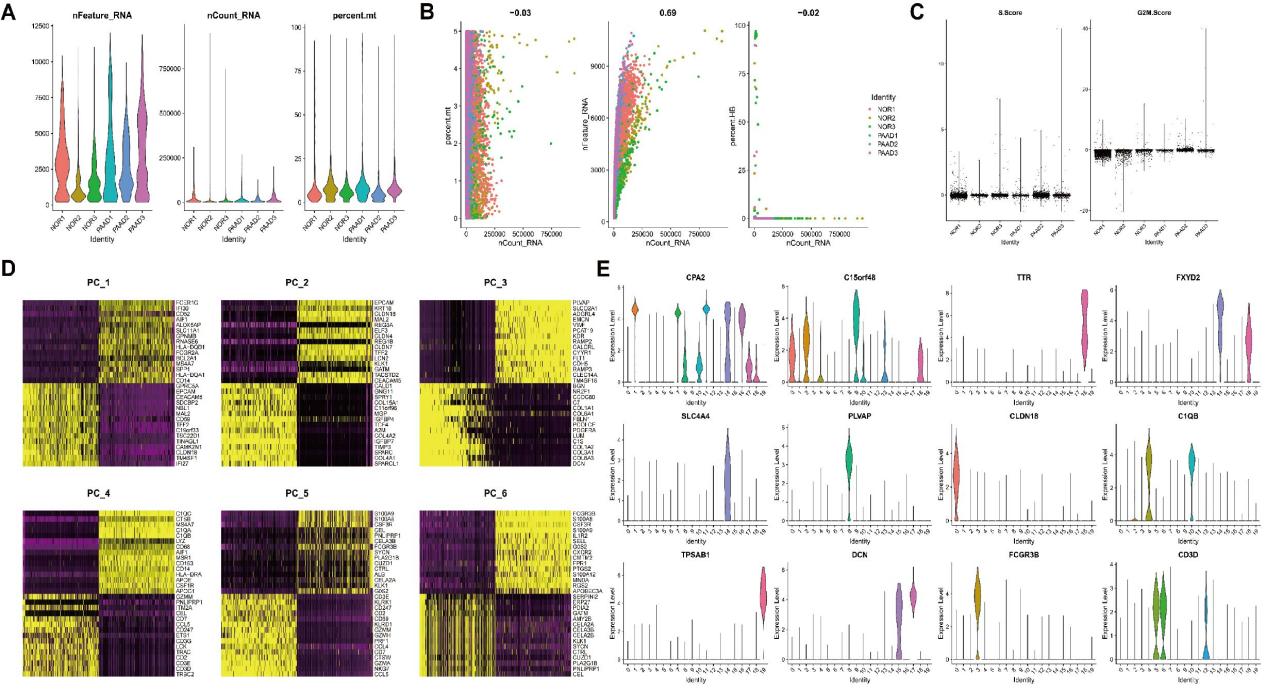
**Figure S1. Quality control and PCA dimension reduction of scRNA-seq data.**

Note: (A) Violin plots showing the distribution of the number of genes per cell (nFeature_RNA), the number of mRNA molecules (nCount_RNA), and the percentage of mitochondrial genes (percent.mt) in scRNA-seq data (N=6); (B) Scatter plots illustrating the correlation between filtered data nCount_RNA and percent.mt, nCount_RNA and nFeature_RNA, and nCount_RNA and percent.HB (N=6); (C) Cell cycle states of each cell in scRNA-seq data, where S.Score represents the S phase and G2M.Score represents the G2M phase (N=6); (D) Heatmaps displaying the expression profiles of the top 20 associated genes in PCA components PC_1 to PC_6, with yellow indicating upregulation and purple indicating downregulation (N=6); (E) Expression patterns of known lineage-specific marker genes in different clusters of normal adjacent tissue samples (Normal, N=3) and PAAD samples (Tumor, N=3).


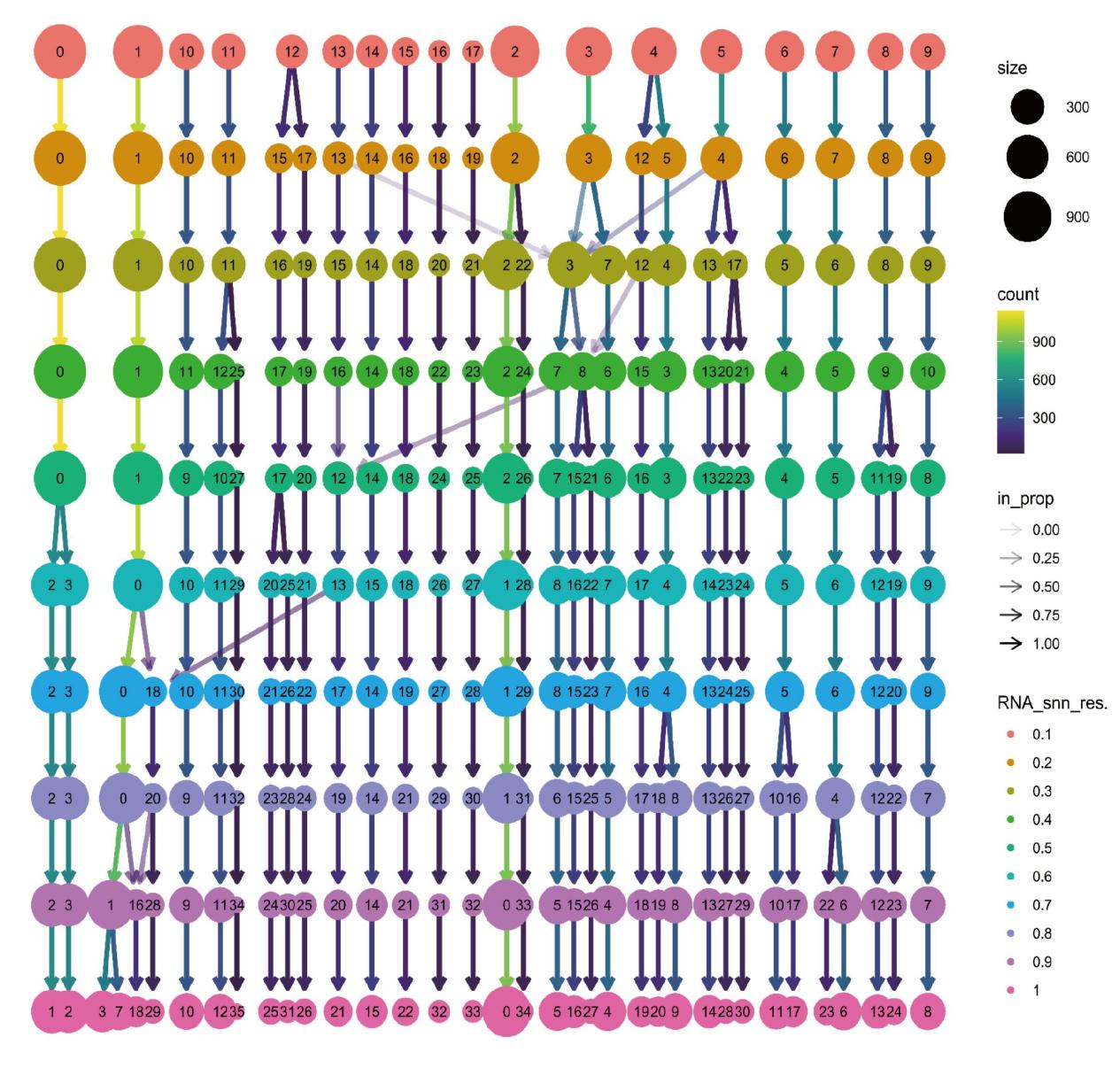
**Figure S2. TSNE clustering dendrogram of scRNA-seq data.**


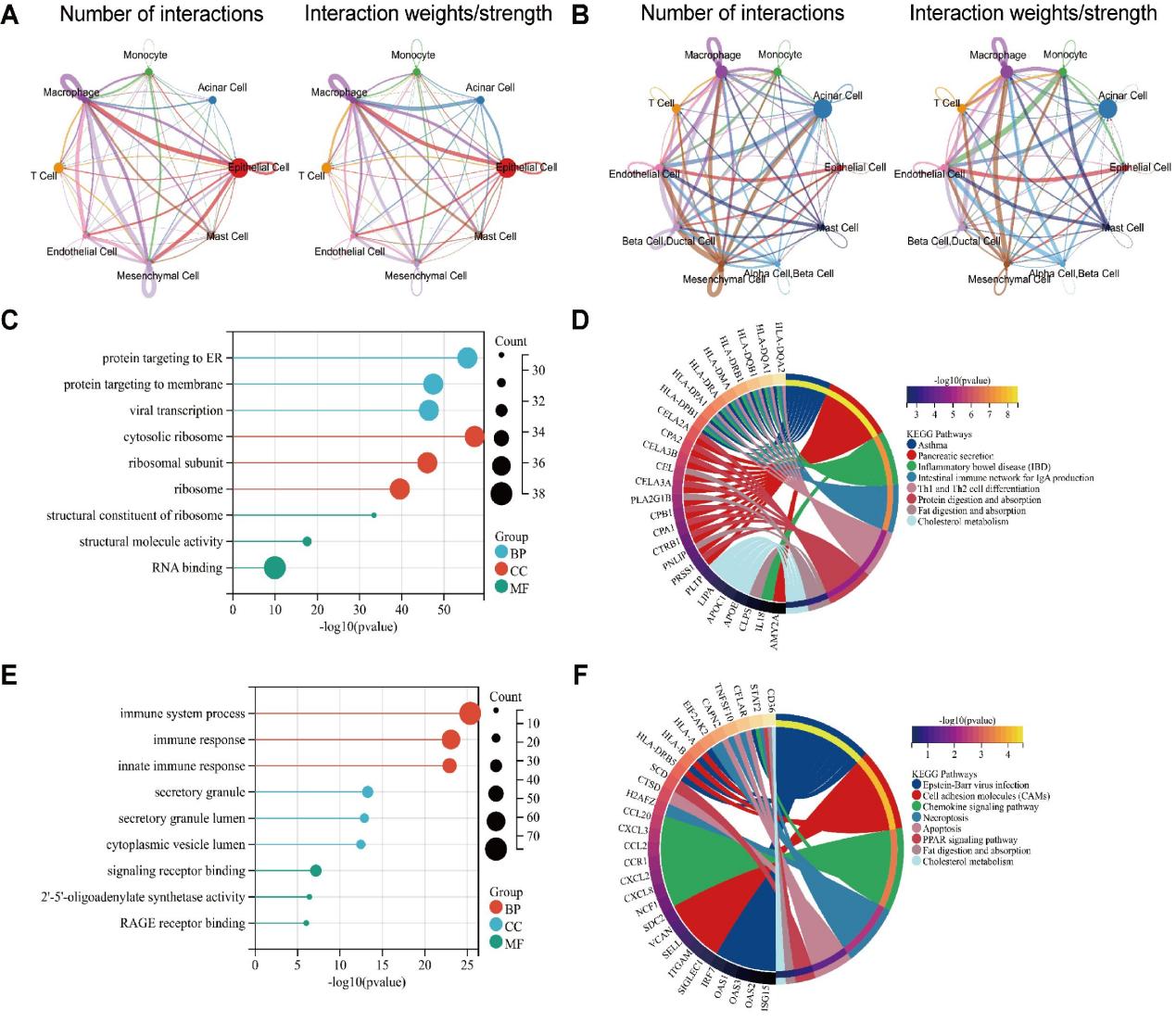
**Figure S3. Enrichment analysis of cell communication and differentially expressed genes.**

Note: (A) Circular plot of cell communication in normal adjacent tissue samples, where the thickness of the lines represents the number of pathways and the strength of the interactions (N=3); (B) Circular plot of cell communication in PAAD samples, where the thickness of the lines represents the number of pathways and the strength of the interactions (N=3); (C) Bar chart showing the GO enrichment of highly expressed genes in PAAD tissue samples, with blue, red, and green representing biological process (BP), cellular component (CC), and molecular function (MF), respectively; (D) Circular plot displaying the KEGG enrichment of highly expressed genes in PAAD samples; (E) Bar chart showing the GO enrichment of lowly expressed genes in PAAD samples, with blue, red, and green representing BP, CC, and MF, respectively; (F) Circular plot displaying the KEGG enrichment of lowly expressed genes in PAAD samples.


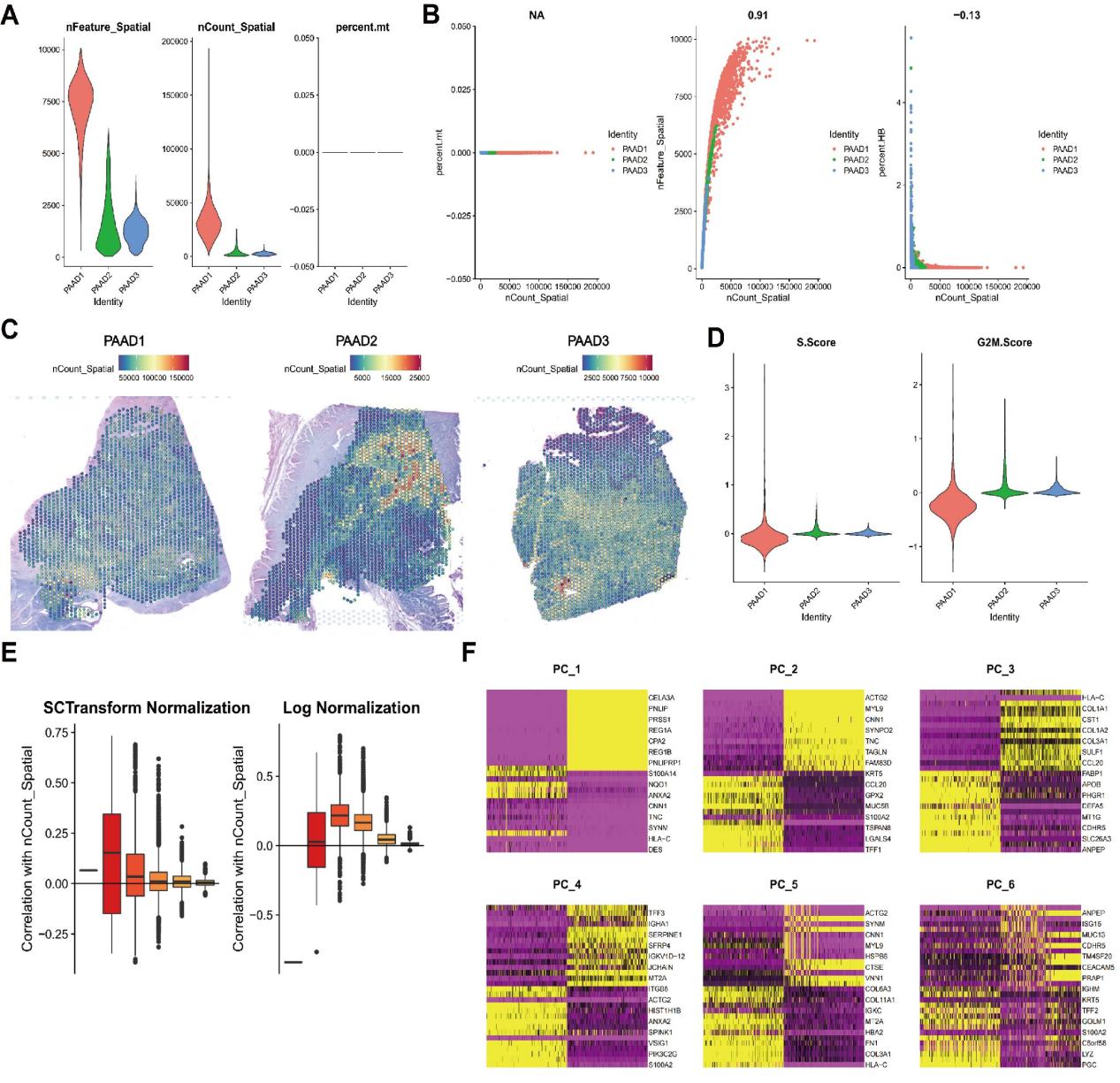
**Figure S4. Quality control and PCA dimension reduction of ST data.**

Note: (A) Violin plots showing the distribution of the number of genes per cell (nFeature_Spatial), the number of mRNA molecules (nCount_Spatial), and the percentage of mitochondrial genes (percent.mt) in ST data (N=3); (B) Scatter plots illustrating the correlation between ST data nCount_Spatial and percent.mt, nCount_Spatial and nFeature_Spatial, and nCount_Spatial and percent.HB (N=3); (C) Distribution of nCount_Spatial on tissue sections in ST data, with a more intense red color indicating higher expression levels of nCount_Spatial for that spot (N=3); (D) Cell cycle states of each cell in ST data, where S.Score represents the S phase and G2M.Score represents the G2M phase (N=3); (E) Standardization results graph of SCTransform and LogNormalize in ST data (N=3); (F) Heatmaps displaying the expression profiles of the top 20 associated genes in PCA components PC_1 to PC_6, with yellow indicating upregulation and purple indicating downregulation (N=3).


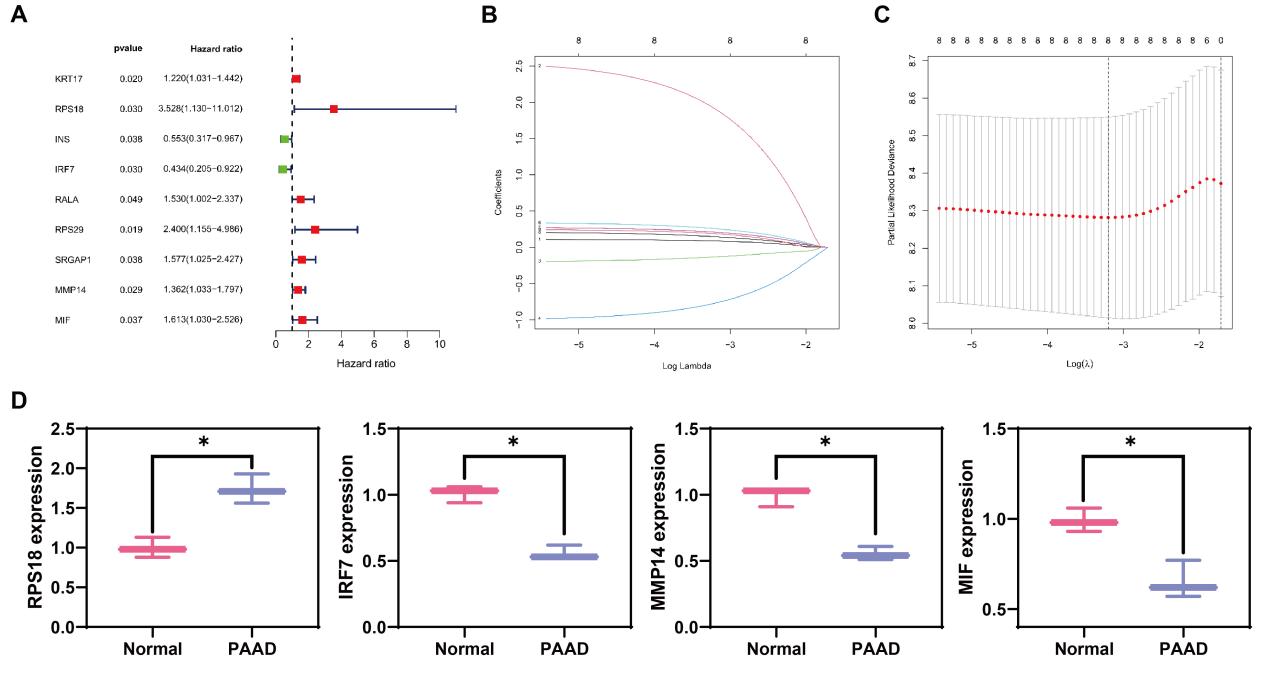
**Figure S5. Construction of the Cox model for the gene and its expression profile.**

Note: (A) The forest plot of univariate Cox analysis of differentially expressed genes related to lipid metabolism in macrophages based on the TCGA database shows 9 prognosis-related genes (red indicating positive correlation, green indicating negative correlation). (B) Distribution of lasso coefficients, with each line representing a gene and the vertical coordinate indicating the coefficient of the gene. (C) Lasso regression model plot, with the vertical coordinate representing cross-validation error and the minimum error point indicating the optimal number of genes in the model. (D) Expression levels of RPS18, IRF7, MMP14, and MIF in macrophages of single-cell sequencing samples, where Normal represents normal adjacent tissue (N=3) and PAAD represents PAAD tissue (N=3). * indicates *P* < 0.05 compared to the Normal group.


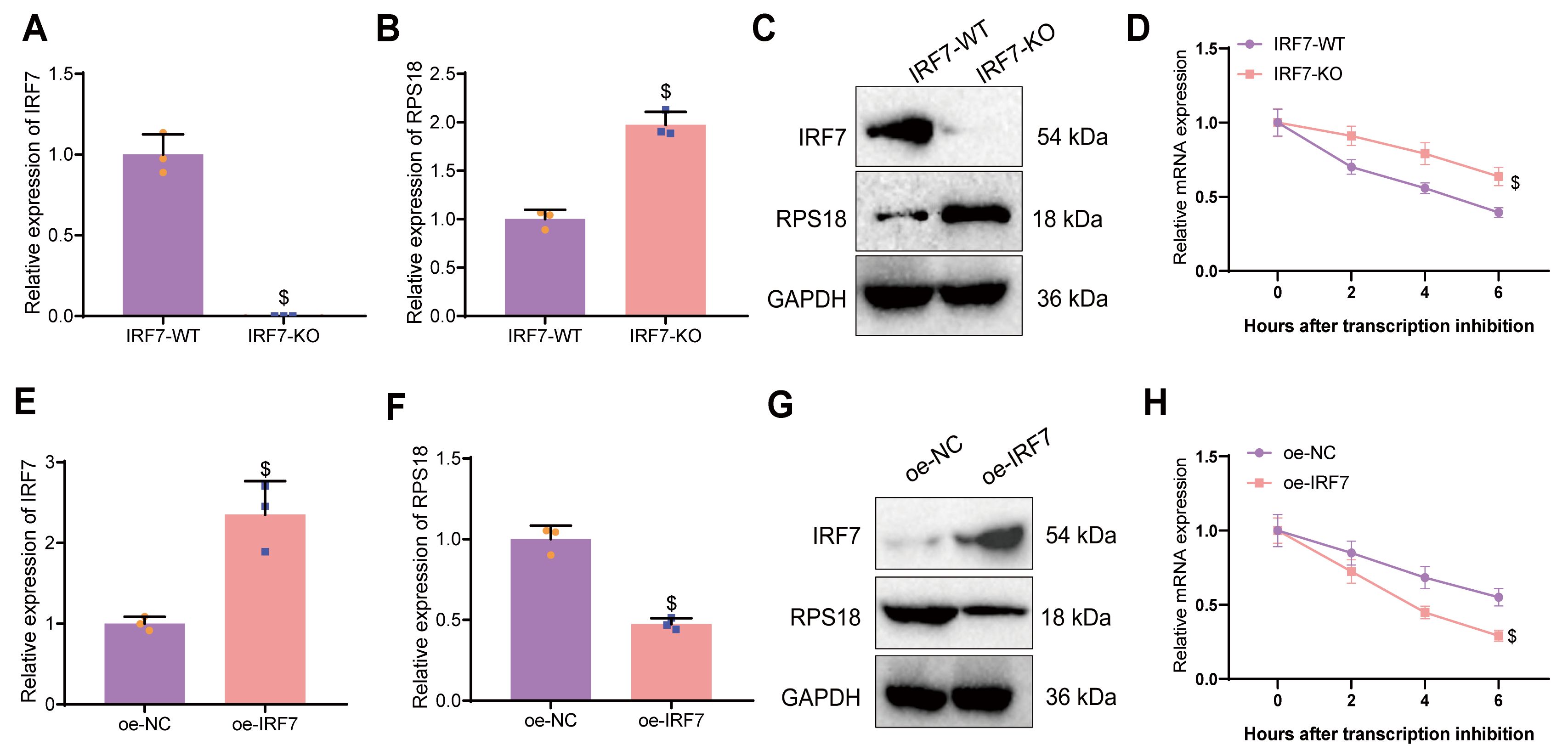


**Figure S6. Validation of IRF7 knockout and overexpression efficiency and RPS18 expression levels.**

Note: (A-B) RT-qPCR analysis of the expression levels of IRF7 and RPS18 in CRISPR/Cas9 gene-edited IRF7-KO cells; (C) Western blot analysis of the expression levels of IRF7 and RPS18 in CRISPR/Cas9 gene-edited IRF7-KO cells; (D) Determination of the stability of RPS18 mRNA in IRF7-KO cells (n=3); (E-F) Measurement of the expression levels of IRF7 and RPS18 in cells after lentiviral transfection with IRF7 overexpression plasmid using RT-qPCR; (G) Assessment of the expression levels of IRF7 and RPS18 in cells after lentiviral transfection with IRF7 overexpression plasmid using Western blot; (H) Evaluation of the stability of RPS18 mRNA in oe-IRF7 cells (n=3) * indicates *P* < 0.05 compared to IRF7−WT or oe−NCgroup, $ indicates *P* < 0.05 compared to IRF7-WT group. Cell experiments were performed in triplicate.


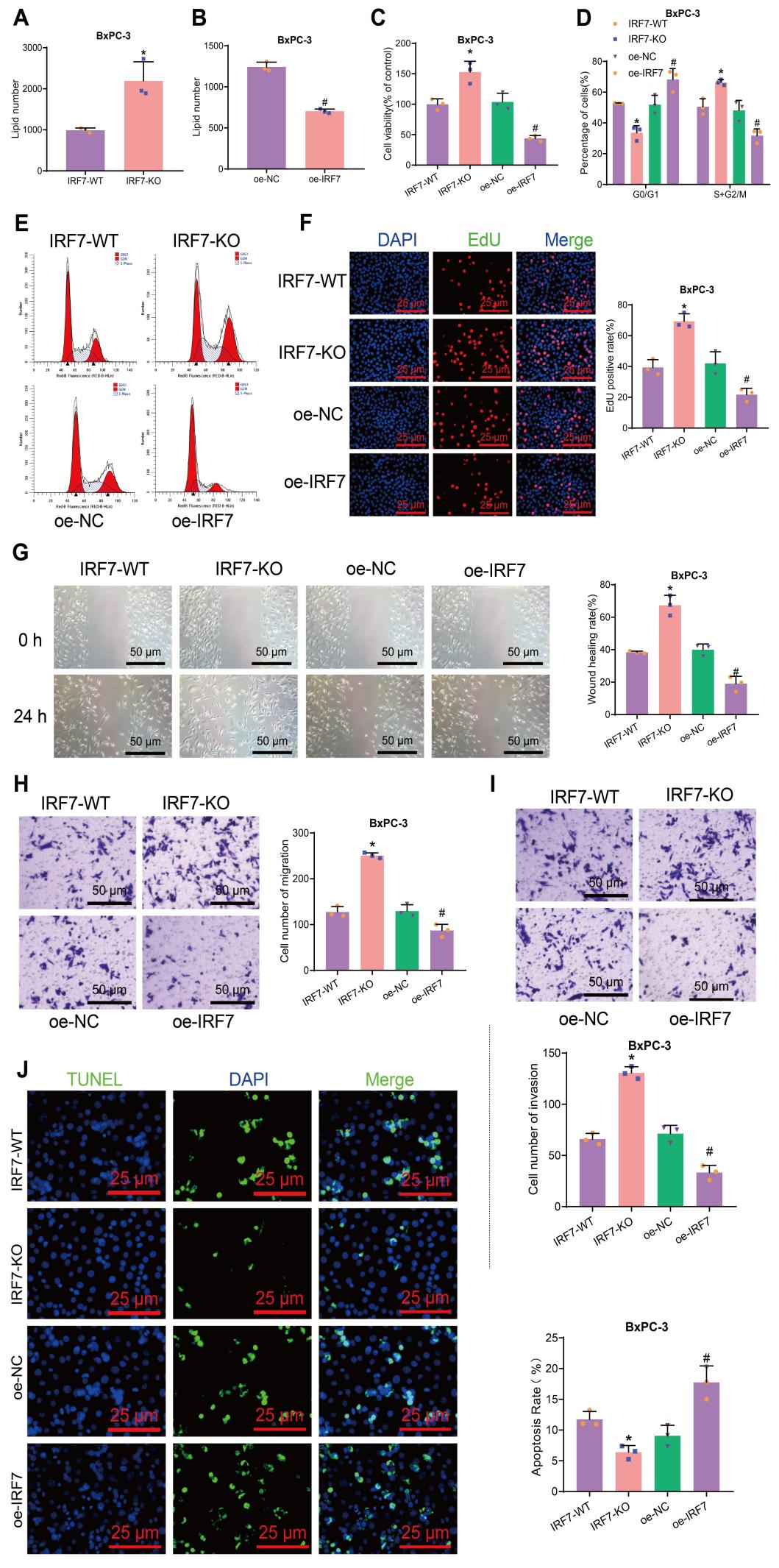


**Figure S7. The Influence of IRF7 on the Biological Function of BxPC-3 Cells.**

Note: (A-B) Metabolomic analysis measuring the total content of lipid metabolites in each group of BxPC-3 cells; (C) MTT assay evaluating the cell viability of each group of BxPC-3 cells; (D) Statistical analysis of flow cytometry results; (E) Flow cytometry analysis examining the cell cycle changes in each group of BxPC-3 cells; (F) EdU experiment assessing the proliferative capacity of each group of BxPC-3 cells (scale bar: 25 μm); (G) Scratch assay measuring the migration ability of each group of BxPC-3 cells (scale bar: 50 μm); (H-I) Transwell assay evaluating the migration and invasion abilities of each group of BxPC-3 cells (scale bar: 50 μm); (J) TUNEL assay measuring the apoptosis rate of each group of BxPC-3 cells (Scale bar=25 μm). * indicates significance compared to the IRF7-WT group with *P* < 0.05, # indicates significance compared to the oe-NC group with *P* < 0.05. All cell experiments were repeated three times.


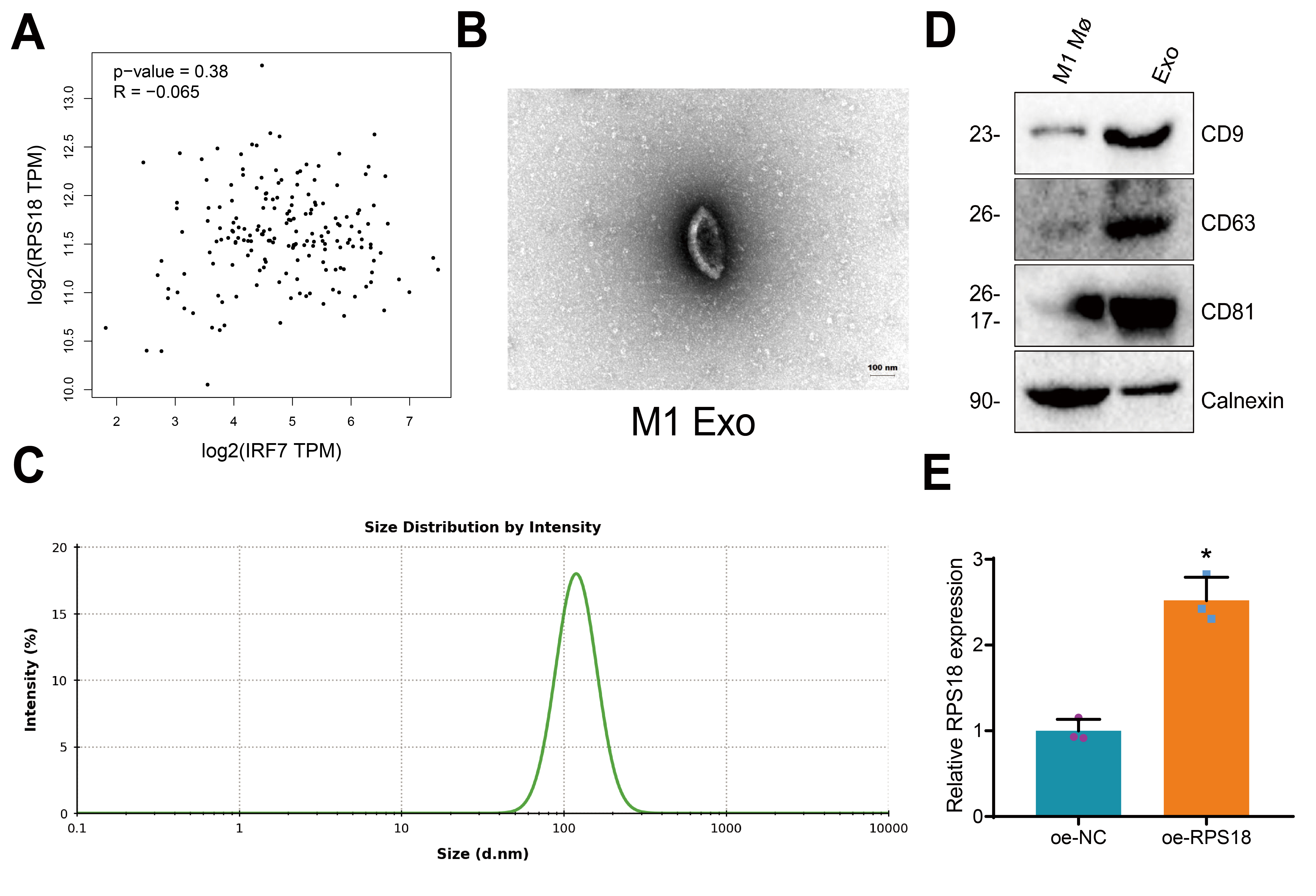


**Figure S8. The identification results and gene correlation analysis of M1-Exos.**

Note: (A) The correlation analysis results between IRF7 and RPS18 in TCGA-PAAD data; (B) TEM observation of the morphological characteristics of M1-Exos, revealing a classic structure of round membrane-bound vesicles (scale bar: 100 nm); (C) Size distribution analysis of M1-Exos via nanoparticle tracking; (D) Western blot detection of the protein expression of CD9, CD63, and CD81 in M1 macrophages and Exos; (E) RT-qPCR assessment of lentiviral transduction efficiency in M1 macrophages. # indicates *P* < 0.05 compared to the oe-NC group, with all cell experiments repeated thrice.


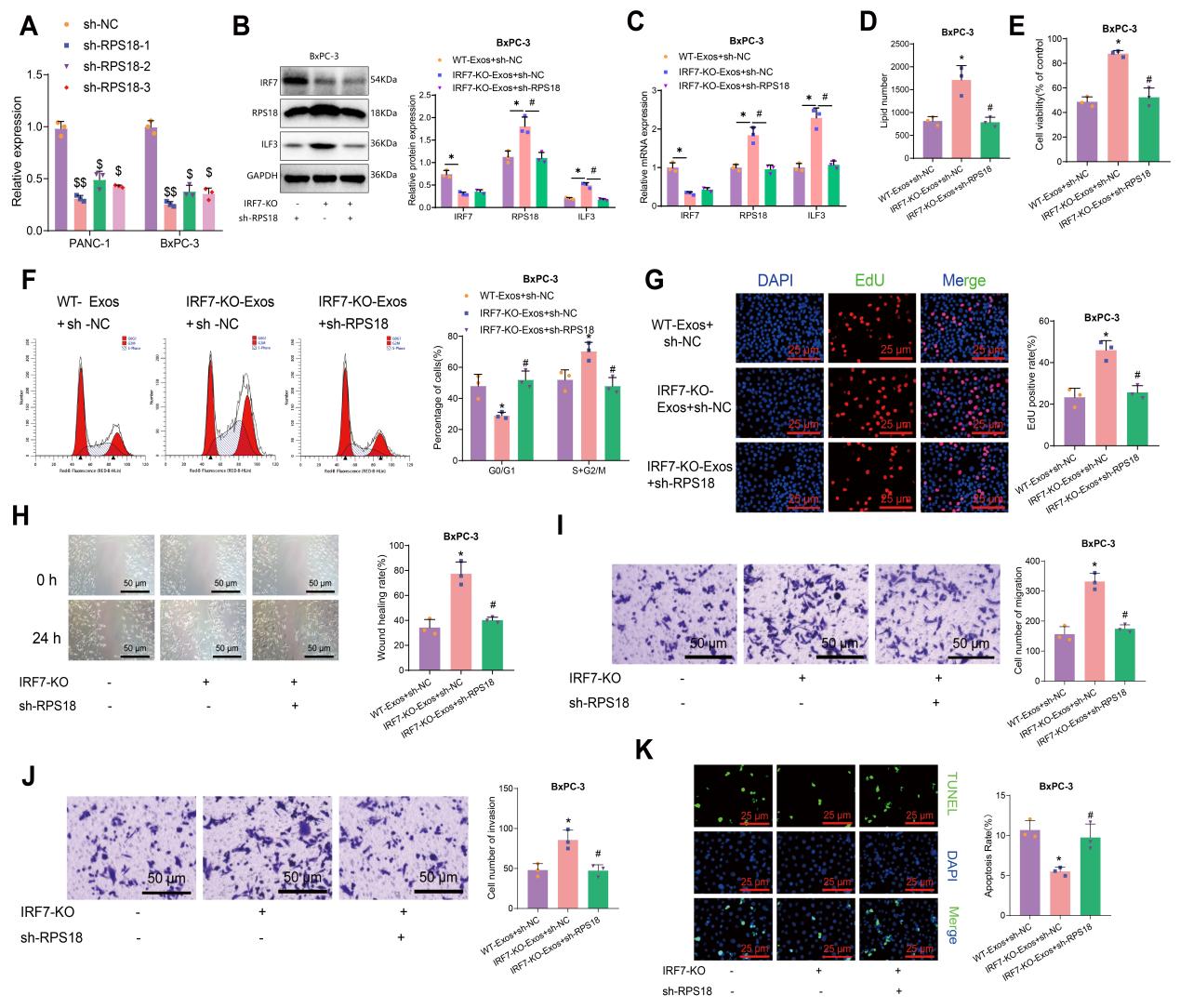


**Figure S9. The Influence of IRF7/RPS18-regulated ILF3 Expression in M1-Exos on the Biological Function of BxPC-3 Cells.**

Note: (A) Assessment of the Silencing Efficiency of 3 RSP18 shRNAs via RT-qPCR; (B) Evaluation of Protein Expression Levels of RPS18, IRF7, and ILF3 in BxPC-3 Cells Across Different Groups Using Western Blot Analysis; (C) Analysis of mRNA Expression Levels of RPS18, IRF7, and ILF3 in BxPC-3 Cells Across Various Groups via RT-qPCR; (D) Metabolomic analysis measuring the total content of lipid metabolites in each group of BxPC-3 cells; (E) MTT assay evaluating the cell viability of each group of BxPC-3 cells; (F) Flow cytometry analysis examining the cell cycle changes in each group of BxPC-3 cells; (G) EdU experiment assessing the proliferative capacity of each group of BxPC-3 cells (scale bar: 25 μm); (H) Scratch assay measuring the migration ability of each group of BxPC-3 cells (scale bar: 50 μm); (I-J) Transwell assay evaluating the migration and invasion abilities of each group of BxPC-3 cells (scale bar: 50 μm); (K) TUNEL assay measuring the apoptosis rate of each group of BxPC-3 cells (Scale bar: 25 μm). * indicates significance compared to the WT-Exos + sh-NC group with *P* < 0.05, # indicates significance compared to the IRF7-KO-Exos + sh-NC group with *P* < 0.05. All cell experiments were repeated three times.


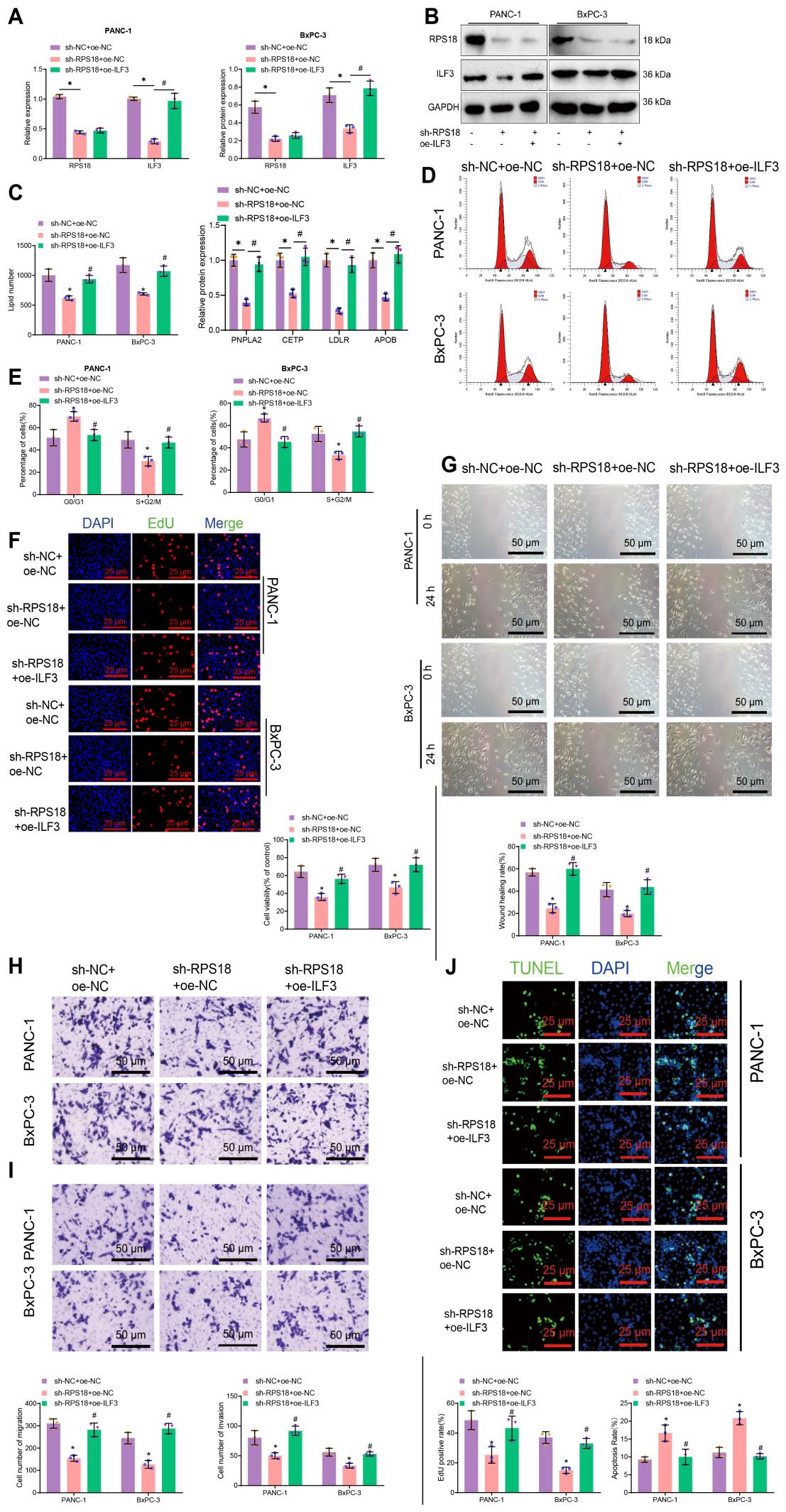


**Figure S10. The Impact of RPS18/ILF3 Axis on the Biological Function of PAAD Cells.**

Note: (A-B) RT-qPCR and Western blot detection of mRNA and protein expression of RPS18, ILF3, PNPLA2, CETP, LDLR, and APOB in each group of cells; (C) Metabolomic analysis and RT-qPCR measuring the total content of lipid metabolites in each group of cells; (D) MTT assay evaluating the cell viability of each group of cells; (E) Flow cytometry analysis examining the cell cycle changes in each group of cells; (F) EdU experiment assessing the proliferative capacity of each group of cells (scale bar: 25 μm); (G) Scratch assay measuring the migration ability of each group of cells (scale bar: 50 μm); (H-I) Transwell assay evaluating the migration and invasion abilities of each group of cells (scale bar: 50 μm); (J) TUNEL assay measuring the apoptosis rate of each group of cells (Scale bar: 25 μm). * indicates significance compared to the sh-NC + oe-NC group with *P* < 0.05, # indicates significance compared to the sh-RPS18 + oe-NC group with *P* < 0.05. All cell experiments were repeated three times.


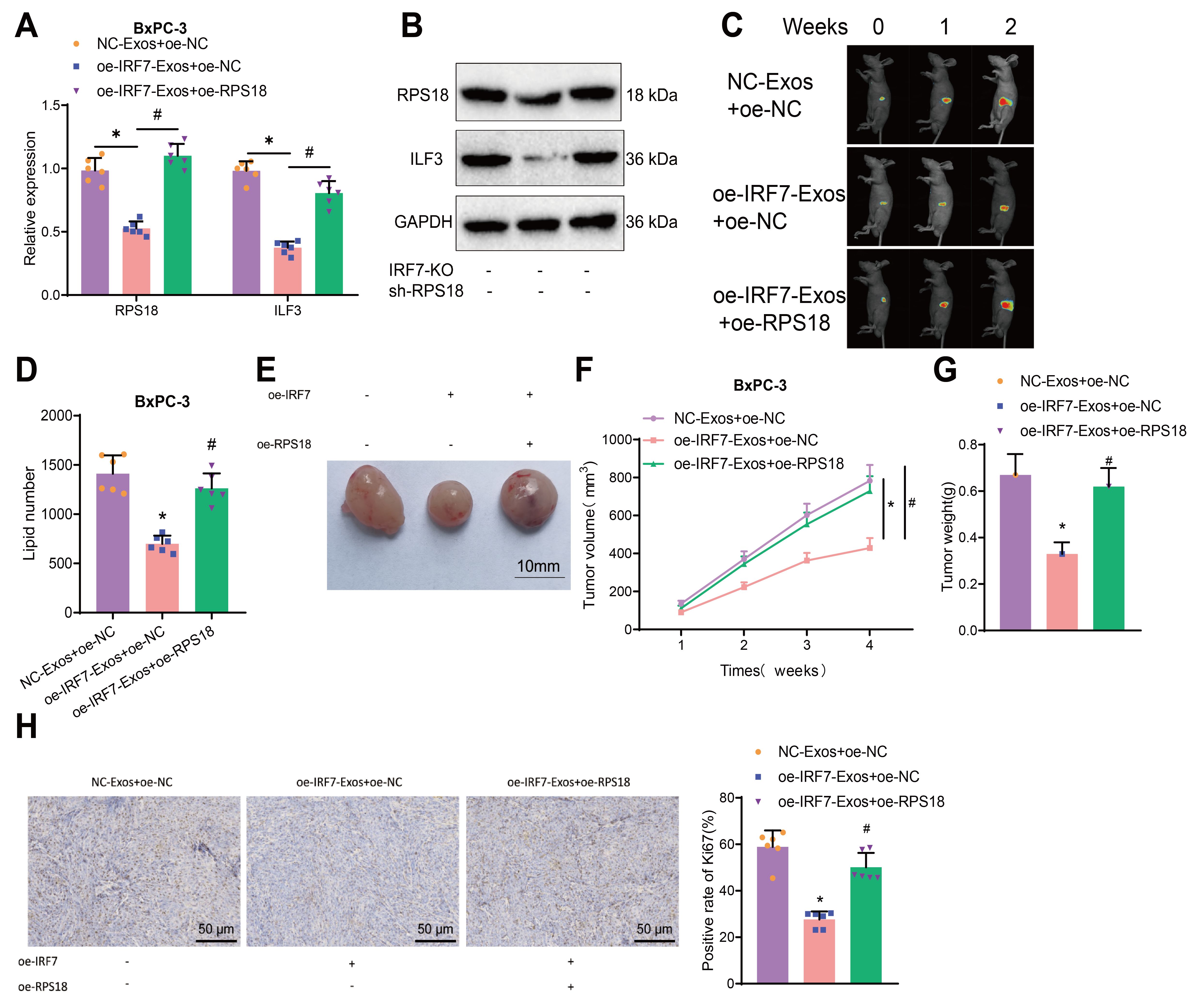


**Figure S11. The Impact of IRF7/RPS18-regulated ILF3 Expression in M1-Exos on Tumor Formation of BxPC-3 Cells *in vivo*.**

Note: (A) RT-qPCR analysis of gene expression levels of RPS18 and ILF3 in the tumor tissues of each mouse group; (B) Western blot detection of protein expression levels of RPS18 and ILF3 in the tumor tissues of each mouse group; (C) Tumor growth monitored using bioluminescence intensity at different time points; (D) Metabolomic analysis measuring the total content of lipid metabolites in the tumor tissues of each mouse group; (E) Morphology of the tumor tissues from each group of mice, with one representative example shown for each group; (F) Tumor growth in each group of mice; (G) Weight of the tumor tissues in each group of mice; (H) Immunohistochemical staining detecting protein expression levels of Ki67 in the tumor tissues of each group of mice (scale bar: 50 μm). * indicates significance compared to the NC-Exos + oe-NC group with P < 0.05, # indicates significance compared to the oe-IRF7-Exos + oe-NC group with *P* < 0.05. Each group consisted of six mice.

Table S1. LASAGNA-Search 2.0 prediction results

| NC_000006.11:33239402-33239901 Homo sapiens chromosome 6, GRCh37.p13 Primary Assembly | | | | RPS18 NM_022551 | | |
| --- | --- | --- | --- | --- | --- | --- |
| Name | Sequence | Position(0-based) | Strand | Score | p-value | E-value |
| IRF-7(M00453) | CAAATGGAAATATGG | 219 | + | 17.33 | 0.000975 | 0.47 |

Table S2. Clinical data of 3 PAAD patients

|  | Number |
| --- | --- |
| Age |  |
| ≤60 | 2 |
| ＞60 | 1 |
| Sex |  |
| Male | 3 |
| Female | 0 |
| Tumor diameter |  |
| < 3 cm | 1 |
| ≥ 3 cm | 2 |
| TNMstage |  |
| I-II | 1 |
| III-IV | 2 |

Table S3. Cell types and markergenes

| Cell Type | Markergenes |
| --- | --- |
| Acinar Cell | REG1B, CTRB1, AMY2A, C15orf48 |
| Alpha Cell, Beta Cell | CRYBA2, TTR, GCG, FXYD2 |
| Beta Cell, Ductal Cell | FXYD2, SLC4A4, CFTR, ANXA4 |
| Endothelial Cell | SPARCL1, PLVAP, A2M, C11orf96 |
| Epithelial Cell | CLDN18, LCN, SPP1, RBPJL |
| Macrophage | C1QB, C1QC, APOE |
| M1 Macrophage | IL1B, CD86 |
| M2 Macrophage | MRC1, CD163 |
| Mast Cell | TPSAB1, TPSB2, CPA3 |
| Mesenchymal Cell | COL3A1, DCN, FN1 |
| Monocyte | FCGR3B, S100A8, PTGS2 |
| T Cell | CD69, PRF1, TRBC2, CD3D |

Table S4. ChIP-qPCR primer sequences

| Gene | Primer Sequence |
| --- | --- |
| RPS18-1 (Human) | F: 5'- GACTCCAGCTGTCCCCTTTC -3' |
|  | R: 5'- AGAGAACAGCGAAGGTTCCG -3' |
| RPS18-2 (Human) | F: 5'- ACCACTTCACCCAACTGCAA -3' |
|  | R: 5'- TTCCGGTGCGCCTTTCTTTA -3' |
| RPS18-3 (Human) | F: 5'- CCCAAAGGGTCTTCCTCAGC -3' |
|  | R: 5'- GTTCCGGGGCTAGTTTGTGT -3' |

F: Forward, R: Reverse

Table S5. shRNA sequences

| shRNAs | Sequence |
| --- | --- |
| sh-RPS18#1 | 5'- GAAGGATGTAAAGGATGGAAA -3' |
| sh-RPS18#2 | 5'- GCGAGTACTCAACACCAACAT -3' |
| sh-RPS18#3 | 5'- CCTTTGCCATCACTGCCATTA -3' |
| sh-NC | 5'- UGAGUAGACUUAACUUUAA -3' |

Table S6. RT-qPCR primer sequences

| Gene | Primer Sequence |
| --- | --- |
| GAPDH (human) | F: 5'-CGGATTTGGTCGTATTGGGC-3' |
|  | R: 5'-TTGACGGTGCCATGGAATTTG-3' |
| IRF7 (human) | F: 5'- GGCTCCTTGGAGAGATCAGC -3' |
|  | R: 5'- CAGCCCAGGCCTTGAAGATG -3' |
| RPS18 (human) | F: 5'- GTGGGCCGAAGATATGCTCA -3' |
|  | R: 5'- TCACACGTTCCACCTCATCC -3' |
| ILF3 (human) | F: 5'- GAACGTAAAACAGCAGGGGC -3' |
|  | R: 5'- TTCGACCTCCATGACGAAGC -3' |
| PNPLA2 (human) | R: 5'- GGCTTCCTCGGCGTCTACTA-3' |
|  | R: 5'- TTTACCAGGTTGAAGGAGGGG-3' |
| CETP (human) | R: 5'- GGCCAAGTCAAGTATGGGTTG -3' |
|  | R: 5'- ACAGACACGTTCTGAATGGAGA-3' |
| LDLR (human) | R: 5'- GAGAGCTTGTGCCGAGATGTG -3' |
|  | R: 5'- CCGCAGTTGTTAGTGCCATCA -3' |
| APOB (human) | R: 5'- TGAGGAGAAGAATCGAACCCT -3' |
|  | R: 5'- CTTGATTTCGTAGAGCAGACAGG -3' |

F: Forward, R: Reverse

Table S7. Details of the first antibody product

| Name | Cat. | Dilution ratio | Manufacturer | Country |
| --- | --- | --- | --- | --- |
| GAPDH | ab9485 | 1: 2500 | Abcam | UK |
| IRF7 | ab238137 | 1: 5000 | Abcam | UK |
| RPS18 | ab224579 | 0.04-0.4 µg/mL | Abcam | UK |
| ILF3 | ab92355 | 1: 1000 | Abcam | UK |
| CD9 | ab307085 | 1: 1000 | Abcam | UK |
| CD63 | ab134045 | 1: 1000 | Abcam | UK |
| CD81 | ab109201 | 1: 1000 | Abcam | UK |
| calnexin | ab22595 | 1 µg/mL | Abcam | UK |
